# The NR4A2/VGF pathway fuels inflammation-induced neurodegeneration via promoting neuronal glycolysis

**DOI:** 10.1172/JCI177692

**Published:** 2024-06-18

**Authors:** Marcel S. Woo, Lukas C. Bal, Ingo Winschel, Elias Manca, Mark Walkenhorst, Bachar Sevgili, Jana K. Sonner, Giovanni Di Liberto, Christina Mayer, Lars Binkle-Ladisch, Nicola Rothammer, Lisa Unger, Lukas Raich, Alexandros Hadjilaou, Barbara Noli, Antonio L. Manai, Vanessa Vieira, Nina Meurs, Ingrid Wagner, Ole Pless, Cristina Cocco, Samuel B. Stephens, Markus Glatzel, Doron Merkler, Manuel A. Friese

**Affiliations:** 1 Institute of Neuroimmunology and Multiple Sclerosis, University Medical Center Hamburg-Eppendorf, Hamburg, Germany.; 2 Department of Biomedical Sciences, NEF-Laboratory, University of Cagliari, Monserrato, Cagliari, Italy.; 3 Department of Pathology and Immunology, Division of Clinical Pathology, Faculty of Medicine, University of Geneva, Geneva, Switzerland.; 4 Protozoa Immunology, Bernhard-Nocht-Institute for Tropical Medicine (BNITM), Hamburg, Germany.; 5 Fraunhofer Institute for Translational Medicine and Pharmacology ITMP, Hamburg, Germany.; 6 Department of Internal Medicine, Fraternal Order of Eagles Diabetes Research Center, University of Iowa, Iowa City, Iowa, USA.; 7 Institute of Neuropathology, University Medical Center Hamburg-Eppendorf, Hamburg, Germany.

**Keywords:** Inflammation, Neuroscience, Multiple sclerosis, Neurodegeneration

## Abstract

A disturbed balance between excitation and inhibition (E/I balance) is increasingly recognized as a key driver of neurodegeneration in multiple sclerosis (MS), a chronic inflammatory disease of the central nervous system. To understand how chronic hyperexcitability contributes to neuronal loss in MS, we transcriptionally profiled neurons from mice lacking inhibitory metabotropic glutamate signaling with shifted E/I balance and increased vulnerability to inflammation-induced neurodegeneration. This revealed a prominent induction of the nuclear receptor NR4A2 in neurons. Mechanistically, NR4A2 increased susceptibility to excitotoxicity by stimulating continuous VGF secretion leading to glycolysis-dependent neuronal cell death. Extending these findings to people with MS (pwMS), we observed increased VGF levels in serum and brain biopsies. Notably, neuron-specific deletion of *Vgf* in a mouse model of MS ameliorated neurodegeneration. These findings underscore the detrimental effect of a persistent metabolic shift driven by excitatory activity as a fundamental mechanism in inflammation-induced neurodegeneration.

## Abstract 2

Inflammation-induced hyperexcitability triggers the neuronal induction of NR4A2, which promotes multiple sclerosis–related neurodegeneration through increased VGF secretion and enhanced glycolysis.

## Graphical abstract

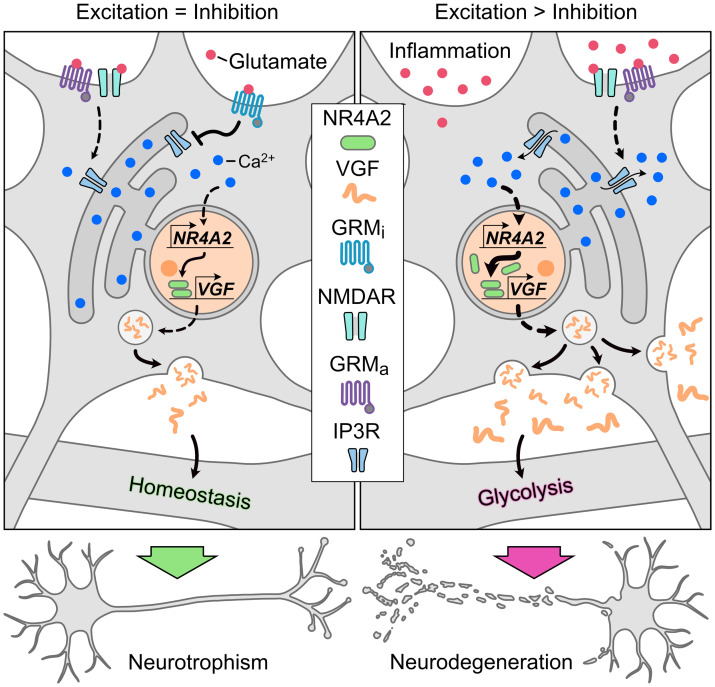

## Introduction

Inflammation-induced neurodegeneration is the principal driver of disease progression and the accumulation of neurological disabilities in people with multiple sclerosis (pwMS) (1, 2), the most prevalent inflammatory disease of the central nervous system (CNS) among young adults (3). MS is believed to begin with autoreactive T cells infiltrating the CNS, leading to demyelination and neuroaxonal damage (4). Concurrently, infiltrating and resident myeloid cells in the CNS sustain a low-grade, “smoldering” inflammation that results in ongoing neuronal loss (5, 6). Although immunomodulatory drugs effectively reduce immune cell infiltration and activation, pwMS continue to experience progressive brain and spinal cord volume loss and neurological deficits, as these treatments do not address the underlying smoldering inflammation (7).

Neurodegeneration in pwMS and its mouse model, the experimental autoimmune encephalomyelitis (EAE), share similarities with other primary neurodegenerative diseases such as Alzheimer’s disease (AD) and Parkinson’s disease (PD) (8), including intracellular protein aggregates (9), mitochondrial dysfunction (10, 11), epigenetic dysregulation (12), and an altered unfolded protein response (UPR) (13). Further, a critical factor is the imbalance in excitatory and inhibitory (E/I) signaling in the CNS (14), which shifts toward hyperexcitability, increasing neuronal vulnerability and loss during neuroinflammation (15, 16). The inflammation-induced chronic hyperexcitability in the CNS arises from the selective loss of inhibitory neurons (17, 18), prolonged exposure to inflammatory cytokines (15), and excessive extracellular glutamate. Glutamate is released from dying cells and infiltrating immune cells and is further amplified by disturbed synaptic integrity, leading to excitotoxic cell death and perpetuating chronic hyperexcitability in CNS inflammation (15, 19).

Increased levels of glutamate have been documented in the cerebrospinal fluid (CSF) and brains of pwMS (20, 21), and histopathological studies support that glutamate-induced transcription factors are induced in pwMS. For example, increased levels of phosphorylated cAMP response element-binding protein (pCREB) and its downstream effector, the orphan nuclear receptor subfamily 4 group A member 2 (NR4A2) (22–24), have been identified in neurons of normal-appearing grey matter (NAGM) and MS lesions (25), providing molecular evidence for chronic hyperexcitability in MS (26). Despite compelling evidence for a E/I imbalance in MS, the impact of continuously upregulated glutamate-induced genes such as NR4A2 on neuronal integrity during CNS inflammation remains unclear.

Similarly, pCREB transcriptionally regulates neurotrophic factors and neuropeptides that have pCREB-binding sites in their promoters, such as brain-derived neurotrophic factor (BDNF), neuronal growth factor (NGF) and VGF nerve growth factor inducible (VGF) (27). In a steady state, these regulate memory formation, synaptic strength, long-term potentiation, and neurogenesis (28–30). Despite their importance for maintaining neuronal homeostasis, their regulation and function in a chronically inflamed CNS during MS are largely unexplored. Additionally, how a chronically shifted E/I balance alters the expression of neurotrophic peptides and affects neuronal resilience is still unclear.

In this context, we previously identified the inhibitory metabotropic glutamate receptor (GRM_i_) 8 (GRM8) as key modulator of vulnerability to glutamate excitotoxicity (25) that limits glutamate-induced calcium release from the endoplasmic reticulum (ER). Accordingly, *Grm8*-deficient neurons display hyperexcitability, making them more susceptible to glutamate-induced cell death, and *Grm8*-deficient mice are more prone to neuronal loss in EAE (25). Thus, *Grm8* deficiency mimics the hyperexcitable phenotype observed in pwMS and EAE and can be utilized to understand the pathways that render neurons susceptible to a chronically skewed E/I balance during CNS inflammation. Therefore, a more profound understanding of the neuronal response to an inflammatory milieu and the signaling networks activated by sustained glutamate exposure is warranted to amend maladaptive cellular alterations. This will then inform the design of treatment strategies and biomarkers for neurodegeneration in MS, with the aim of ultimately restoring homeostasis.

Here, we set out to investigate the molecular pathways that are initiated by imbalanced glutamate signaling during CNS inflammation in MS. Using *Grm8*-deficient mice, we explored the neuronal susceptibility to CNS inflammation due to a shifted E/I balance (25) and found that NR4A2 is induced in neurons. This induction of NR4A2 caused glycolysis-dependent cell death through chronic secretion of VGF by neurons. Accordingly, we observed increased VGF levels in the plasma of pwMS and in neurons from brain biopsies of pwMS. In the preclinical EAE model, *Vgf*-deficient mice show less neurodegeneration. These results support that persistent neurotrophic signaling via VGF, driven by chronic hyperexcitability, is maladaptive in CNS inflammation and accelerates neuronal loss.

## Results

### NR4A2 is induced in Grm8-deficient neurons.

**Figure 1:**
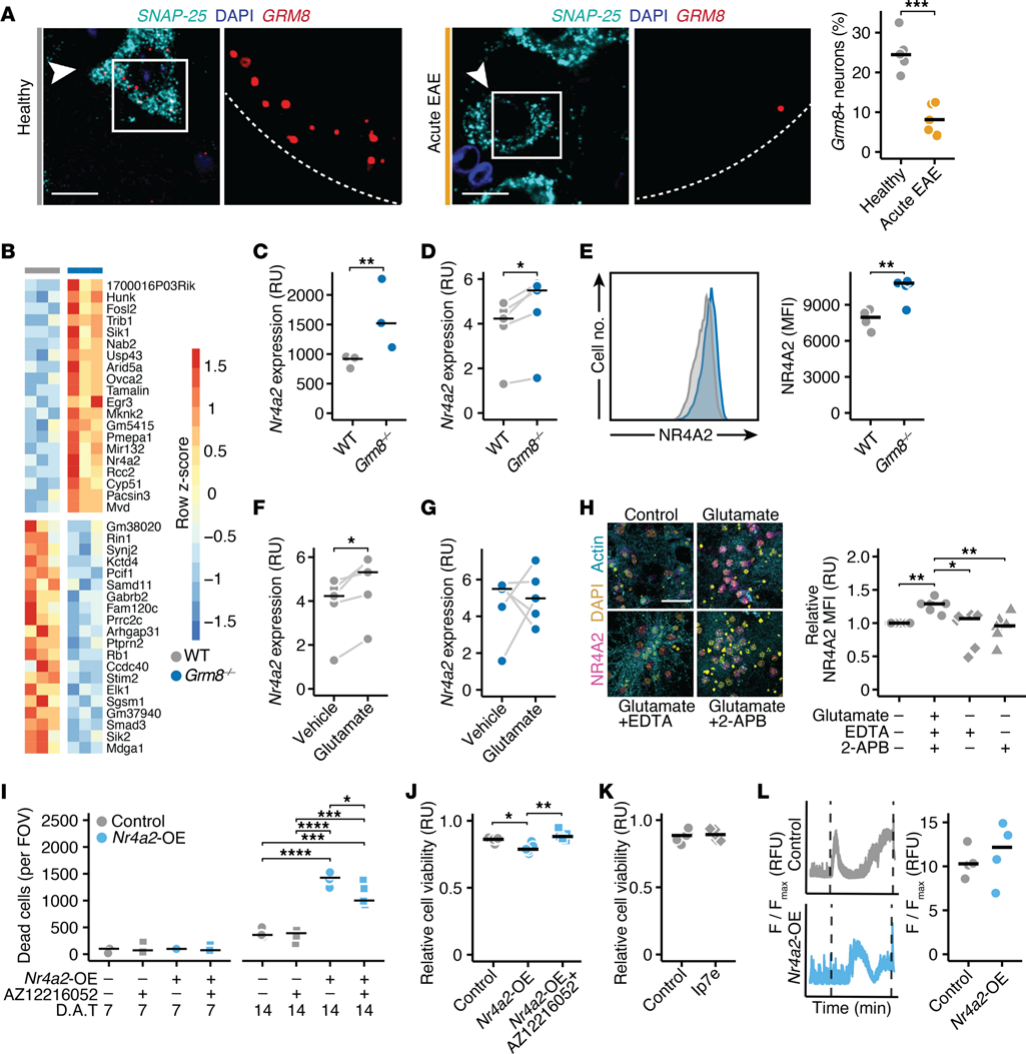
NR4A2 is induced in *Grm8*-deficient neurons. (**A**) The number of *Grm8*^+^ neurons assessed by RNAScope in cervical spinal cords of healthy and EAE mice 15 days after immunization (*n* = 5). Scale bar: 20 μm. (**B**) Heatmap of top 15 up and downregulated genes in NeuN^+^ cortical nuclei of *Grm8*^–/–^ compared with WT mice (*n* = 3). (**C**) Relative *Nr4a2* expression in NeuN^+^ cortical nuclei of *Grm8*^–/–^ and WT mice in relative units (RU). (**D**) *Nr4a2* expression (RU) in WT and *Grm8*^–/–^ primary cortical neurons (PCNs; *n* = 5). Paired *t* test was performed. (**E**) NR4A2 mean fluorescence intensity (MFI) in cortical neuronal nuclei estimated by flow cytometry of WT (*n* = 4) and *Grm8*^–/–^ mice (*n* = 5). (**F** and **G**) *Nr4a2* expression (RU) in WT (**F**) and *Grm8*^–/–^ (**G**) PCNs after glutamate stimulation (*n* = 5). Paired *t* test was performed. (**H**) NR4A2 MFI in PCNs that were treated with glutamate and vehicle, 2 mM EDTA or 50 μM 2-APB (*n* = 6). Scale bar: 20 μm. (**I**) Dead cells per field of view (FOV) of control (mScarlet) or *Nr4a2*-overexpressing PCNs 7 days after transduction (D.A.T.; left) and 14 D.A.T. (right). PCNs were treated with vehicle or 1 μM AZ12216052 every other day (*n* = 5). (**J** and **K**) Viability (RU) of control (mScarlet) or *Nr4a2*-overexpressing PCNs that were exposed to glutamate and vehicle or 1 μM AZ12216052 (*n* = 5, **J**) or 50 nM Ip7e (*n* = 5; **K**). (**L**) Calcium traces and somatic calcium accumulation in glutamate-treated control (mScarlet) or *Nr4a2*-overexpressing PCNs (*n* = 4). Points represent individual experiments, additionally, the mean is shown. If not stated otherwise, unpaired *t* test with FDR correction for multiple comparisons was used. *P < 0.05, **P < 0.01, ***P < 0.001, ****P < 0.0001.

Recently, we identified the neuronally expressed GRM8 as a decisive protector of hyperexcitability and neurodegeneration in neuronal cultures and during CNS inflammation in EAE mice (25). Having demonstrated that GRM8 is expressed in neurons of both people in the control group and pwMS (25), we first probed the expression of *Grm8* transcripts in motoneurons of the cervical spinal cord in healthy and EAE mice. We found that *Grm8*-positive neurons were significantly reduced in EAE (Figure 1A), indicating a loss of inhibitory glutamate signaling during CNS inflammation. Given that neuronal *Grm8* deficiency results in a chronic hyperexcitable state that renders neurons more susceptible to inflammation-induced neurodegeneration (25), we used *Grm8*-deficient (*Grm8^–/–^*) mice to investigate how chronic neuronal loss is mediated by hyperexcitability during neuroinflammation. We isolated NeuN-positive neuronal nuclei from cortices of WT and *Grm8^–/–^* mice by flow cytometry sorting (Supplemental Figure 1A; supplemental material available online with this article; https://doi.org/10.1172/JCI177692DS1) and sequenced their transcripts (Figure 1B and Supplemental Figure 1B). Among the 108 differentially expressed genes, NR4A2 stood out as one of the most highly upregulated genes (Figure 1C). As NR4A2 is a transcription factor belonging to the steroid hormone receptor class associated with autoimmune, neurodevelopmental, and neurodegenerative diseases (31–33), and is increased in neurons of pwMS (26), it qualified as a potential regulator of neuronal integrity. Indeed, we validated its increased mRNA expression in neuronal cultures isolated from *Grm8^–/–^* mice (Figure 1D). Next, we measured the NR4A2 protein by flow cytometry in isolated nuclei from the cortex. NR4A2 was expressed more highly in neuronal than in nonneuronal nuclei (Supplemental Figure 1, C and D), and, in line with our sequencing data, we found increased NR4A2 protein levels in neuronal nuclei of *Grm8^–/–^* mice (Figure 1E). Since we hypothesized that the increased expression of NR4A2 in *Grm8^–/–^* neurons was due to excessive glutamatergic signaling, we next tested whether glutamate could induce NR4A2 expression. WT neurons that were exposed to glutamate showed an increased *Nr4a2* expression compared with vehicle-treated neurons (Figure 1F). Notably, this was not the case for *Grm8^–/–^* neurons, which already had increased baseline levels of *Nr4a2* and did not show an additional induction after glutamate exposure (Figure 1G). Further, we were able to inhibit glutamate-induced NR4A2 upregulation in WT neurons by chelating calcium or by blocking IP3R-dependent calcium release from the ER (Figure 1H). Together, these results indicate that NR4A2 is upregulated as a consequence of an E/I imbalance with hyperexcitability.

### Chronic NR4A2 expression induces neurotoxicity.

Since chronic neuroinflammation continuously disturbs the E/I balance, we next asked whether prolonged NR4A2 expression impacts neuronal cell viability. Therefore, we constructed lentiviral *Nr4a2* overexpression constructs and respective mScarlet control vectors. Lentiviral transduction of neuronal cultures with NR4A2 overexpression constructs increased *Nr4a2* transcripts by approximately 6-fold and NR4A2 protein levels by 2-fold (Supplemental Figure 1, E and F). Chronic overexpression of NR4A2 over 14 days resulted in increased cell death compared with mScarlet control transduction, which was partially inhibited by daily treatment with the allosteric GRM8 agonist AZ12216052 (Figure 1I). Further, NR4A2-overexpressing neurons were more vulnerable to glutamate-induced toxicity, which could again be mitigated by elevating GRM8 activity using AZ12216052 (Figure 1J). In contrast, acute pharmacological activation of NR4A2 with the potent compound Ip7e (34) did not render the neurons more susceptible to glutamate toxicity (Figure 1K), underscoring that continuous NR4A2 signaling was required to increase neuronal vulnerability. Since we found that NR4A2 was upregulated in *Grm8^–/–^* neurons and previously described that GRM8 activity ameliorates glutamate-induced neuronal calcium accumulation (25), we tested whether neuronal calcium homeostasis was regulated by NR4A2. However, we found no differences in baseline calcium activity (Supplemental Figure 1G) or in calcium accumulation after glutamate stimulation in NR4A2-overexpressing neurons compared with control neurons (Figure 1L). Thus, we concluded that mechanisms other than glutamate-induced calcium accumulation underlie the neuronal vulnerability exerted by NR4A2.

### VGF mediates NR4A2-induced neuronal vulnerability.

**Figure 2:**
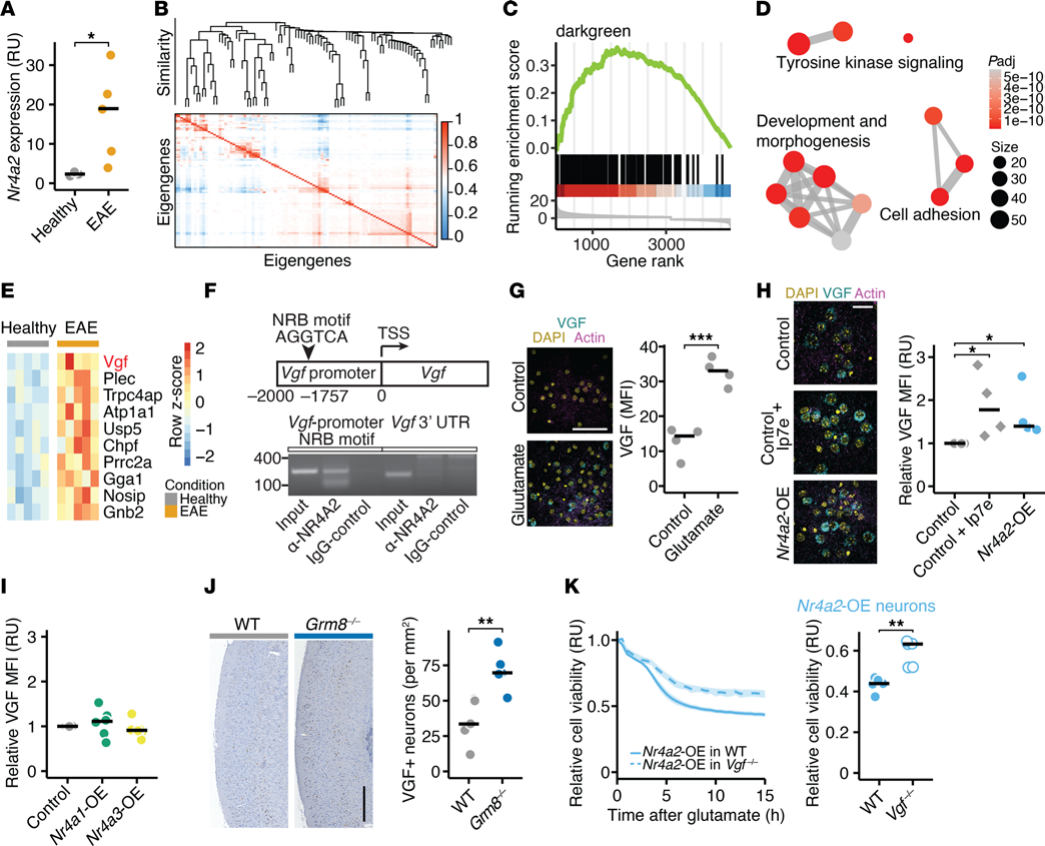
NR4A2-induced VGF mediates neuronal susceptibility to excitotoxicity. (**A**) *Nr4a2* mRNA expression in NeuN^+^ nuclei sorted from spinal cords of healthy mice (*n* = 3) and acute EAE mice (*n* = 5) in relative units (RU). (**B**) Eigengene correlation matrix and dendrogram of weighted gene correlated network analysis (WGCNA) with 502 neuron-specific transcriptome data sets. (**C**) Enrichment analysis of the inflamed neuronal signature (ranked gene list retrieved from ref. 9) in module “darkgreen”. (**D**) GO term biological process enrichment analysis of the module “darkgreen”. Size shows number of genes of GO terms, color shows significance. (**E**) Heatmap of top 10 genes from the module “darkgreen,” which are differently expressed in neurons during EAE. (**F**) Chromatin immunoprecipitation (ChIP) was performed using an antibody against NR4A2 and an IgG control from 3 pooled mouse cortices. PCR primers were designed to amplify approximately 100 nucleotides flanking the canonical nuclear receptor binding (NRB) motif in the mouse *Vgf* promoter (left) or the 3′ untranslated region of *Vgf* as control (right). (**G**) VGF MFI in neuronal cultures after vehicle or glutamate stimulation (*n* = 4 per group). Scale bar: 50 μm. (**H** and **I**) Relative VGF MFI in neurons that overexpress mScarlet (controls), *Nr4a2* or were exposed to 50 nM Ip7e (**H**; *n* = 4), and neurons that overexpress *Nr4a1* or *Nr4a3* (**I**; *n* = 6). Data is normalized to mScarlet-overexpressing control neurons. Scale bar: 20 μm. (**J**) VGF^+^ neurons in cortices of WT and *Grm8^–/–^* mice (*n* = 5). Scale bar: 300 μm. (**K**) Cell viability (RU) of WT and *Vgf^–/–^* neurons that overexpress *Nr4a2* and were exposed to glutamate (*n* = 5). Points represent individual experiments, additionally mean is shown. If not stated otherwise, unpaired *t* test with FDR correction for multiple comparisons was used. *P < 0.05, **P < 0.01, ***P < 0.001.

Next, we aimed to identify how elevated NR4A2 activity contributes to neuronal vulnerability during inflammation. We analyzed neuronal *Nr4a2* expression in EAE and found that it was induced in neuronal nuclei of spinal cords in EAE mice, further providing evidence for chronic hyperexcitability that might drive neuronal demise (Figure 2A). To identify the downstream network of NR4A2, we performed weighted gene correlation network analysis (WGCNA) of 502 publicly available neuron-specific transcriptome data sets from in vitro and in vivo models of neurodegeneration and neuroinflammation (Figure 2B; all data sets are provided in Supplemental Table 1). We identified 80 modules that included more than 5 genes and less than 1,000 genes, 23 of which included *Nr4a2*. To prioritize NR4A2-dependent modules that might influence neuronal viability during neuroinflammation, we compiled a neuronal signature in response to inflammation from our previously published neuronal translatome profiling of EAE mice (9). We used this signature and ran an enrichment analysis for all *Nr4a2*-containing modules. We identified 3 modules that were positively enriched and 5 modules that were negatively enriched in neurons from EAE mice (Supplemental Figure 2A; differentially expressed genes of positively enriched modules and top negatively enriched modules are provided in Supplemental Table 2). Since *Nr4a2* is significantly upregulated in neurons of EAE mice (Figure 2A), we focused on the *Nr4a2*-containing module “darkgreen”, which was most significantly positively enriched in EAE mice (Figure 2C; normalized enrichment score [NES] = 2.2, *P*_adj_ = 1 × 10^–6^). By performing gene ontology (GO) term enrichment analysis, we found that the darkgreen module contained genes that regulate development, growth, and morphogenesis (Figure 2D). To detect key regulators of this module in EAE, we overlapped its defining genes with all differentially expressed genes in inflamed motoneurons during EAE and identified *Vgf* as the most significantly induced gene (Figure 2E). We confirmed direct binding of NR4A2 to the nuclear receptor binding motif of the *Vgf* promoter by chromatin immunoprecipitation (ChIP) in the mouse cortex (Figure 2F). Similar to NR4A2, VGF protein levels were upregulated in neurons exposed to glutamate excitotoxicity as measured by immunofluorescence (Figure 2G). Notably, prolonged NR4A2 expression in neurons or acute exposure to the pharmacological NR4A2 agonist Ip7e directly increased VGF levels in neurons (Figure 2H). In contrast, lentiviral overexpression of the structurally closest nuclear receptor transcription factors NR4A1 and NR4A3 did not induce VGF levels (Figure 2I and Supplemental Figure 2B). Additionally, we observed an increased number of VGF-positive neurons in the cortices of *Grm8^–/–^* mice, further supporting that VGF is continuously induced when inhibitory glutamatergic signaling is impaired (Figure 2J). To directly assess the causal link between NR4A2-induced VGF expression and heightened neuronal vulnerability, we expressed NR4A2 or mScarlet in both WT and *Vgf*-deficient neurons (Supplemental Figure 2C), followed by glutamate exposure. While we detected no differences in baseline cell viability between *Vgf*-deficient and WT neurons (Supplemental Figure 2D), *Vgf*-deficient neurons were protected from glutamate excitotoxicity (Supplemental Figure 2E) and offset NR4A2-mediated vulnerability (Figure 2K). We concluded that prolonged NR4A2 activity directly induced lasting VGF expression that mediated NR4A2-dependent neuronal loss.

### VGF is released by inflamed neurons in EAE and MS.

**Figure 3:**
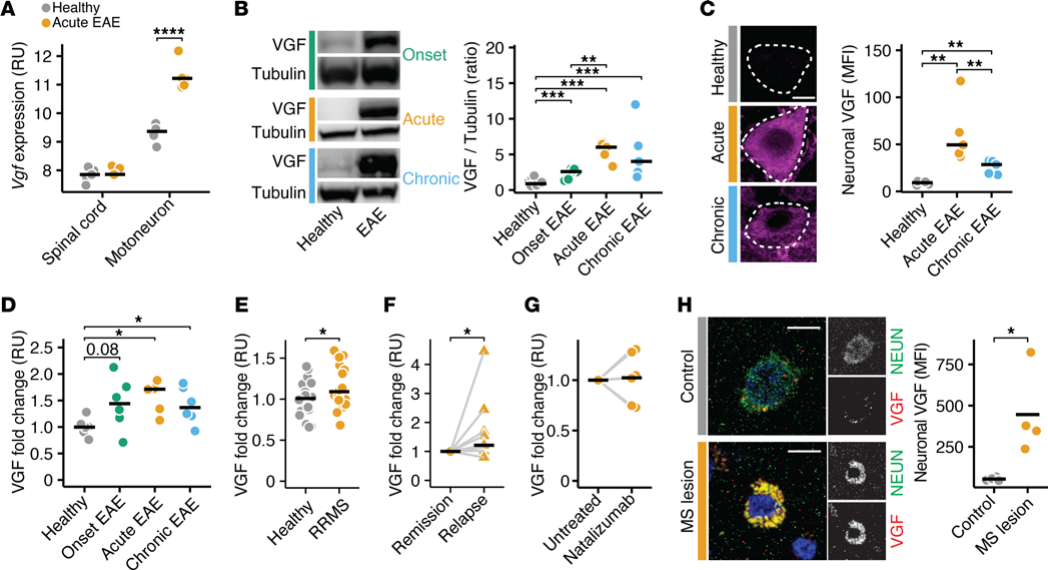
VGF is increased in the CNS and blood of EAE animals and pwMS. (**A**) *Vgf* expression in relative units (RU) in spinal cords and motoneurons of healthy mice and mice undergoing EAE (data was retrieved from ref. 9; *n* = 5 per group). (**B**) VGF protein levels in the spinal cords of healthy mice (*n* = 14) and mice undergoing EAE 10 days (onset, *n* = 6), 15 days (acute, *n* = 5), and 30 days (chronic, *n* = 5) after immunization. (**C**) Neuronal VGF MFI in cervical spinal cords of healthy mice and mice undergoing acute and chronic EAE (*n* = 5 per group). Scale bar: 5 μm. (**D**) Relative fold change of total VGF in the plasma of healthy mice (*n* = 6) and mice undergoing EAE 10 days (onset, *n* = 6), 15 days (acute, *n* = 5), and 30 days (chronic, *n* = 6) after immunization. (**E**) Relative fold change of total VGF in the sera of healthy controls and with pwRRMS (*n* = 20 per group). Controls were age and sex matched. (**F**) Relative fold change of total VGF in sera of pwRRMS during stable disease and acute relapse (*n* = 9). Paired *t* test was used for statistical comparison. (**G**) Relative fold change of total VGF in the sera of pwRRMS before and after treatment with natalizumab (*n* = 6 per group). Paired *t* test was used for statistical comparison. (**H**) Neuronal VGF MFI in brain biopsies of noninflammatory CNS disease controls (controls) and pwMS (*n* = 4 per group). Scale bar: 5 μm. Points represent individual experiments, additionally mean is shown. If not stated otherwise, unpaired *t* test with FDR correction for multiple comparisons was used. *P < 0.05, **P < 0.01, ***P < 0.001, ****P < 0.0001.

After having revealed that VGF caused NR4A2-driven neurodegeneration, we then evaluated whether neuronal expression of VGF was also induced in inflamed neurons in EAE and pwMS. Indeed, in EAE, *Vgf* was strongly upregulated in motoneurons, as identified in our previously profiled neuron-specific translatome (9) (Figure 3A). Accordingly, we found increased VGF levels in whole spinal cords by using immunoblot analysis (Figure 3B) and by IHC in motoneurons during acute and chronic phases of EAE (Figure 3C). Given that VGF is a secreted neuropeptide, we next measured its various cleaved peptides in the serum of EAE animals and calculated the averaged fold changes across all measured cleaved peptides to estimate total circulating VGF levels. Matching our spinal cord results, circulating VGF levels were increased in animals during acute and chronic EAE (Figure 3D; measurements of all individual peptides are shown in Supplemental Figure 3A). To translate our findings to MS, we measured VGF in the blood of untreated pwMS and respective people in the healthy control group. We found that VGF levels were also increased in people with relapsing-remitting MS (pwRRMS) during remission (Figure 3E, measurements of all individual peptides are shown in Supplemental Figure 3B; cohort characteristics are provided in Supplemental Table 3). Moreover, in a second longitudinal cohort of untreated pwRRMS, VGF levels were further increased during an active relapse compared with their remission state (Figure 3F; measurements of all individual peptides are shown in Supplemental Figure 3C), supporting the notion that VGF is released in response to inflammatory challenges of the CNS. Next, we investigated whether VGF release depends on acute inflammation by CNS-infiltrating T cells or on chronic neuroinflammatory processes. Therefore, we compared blood VGF levels in pwMS before and during treatment with the disease-modifying drug natalizumab, which prevents T cell infiltration into the CNS. Notably, we did not detect any intraindividual differences in pwMS before and during natalizumab treatment (Figure 3G; measurements of all individual peptides are shown in Supplemental Figure 3D), indicating that increased VGF blood levels do not reflect acute bouts of CNS inflammation. Finally, we analyzed whether inflamed human neurons similarly upregulate VGF. We stained VGF in brain biopsies obtained from pwMS and controls without a neuroinflammatory disease (cohort characteristics are provided in Supplemental Table 4) and found that VGF was significantly upregulated in neurons from pwMS (Figure 3H). Thus, our data indicate that CNS inflammation induces VGF expression in both EAE and MS.

### Chronic VGF exposure exacerbates excitotoxicity by inducing glycolysis.

**Figure 4:**
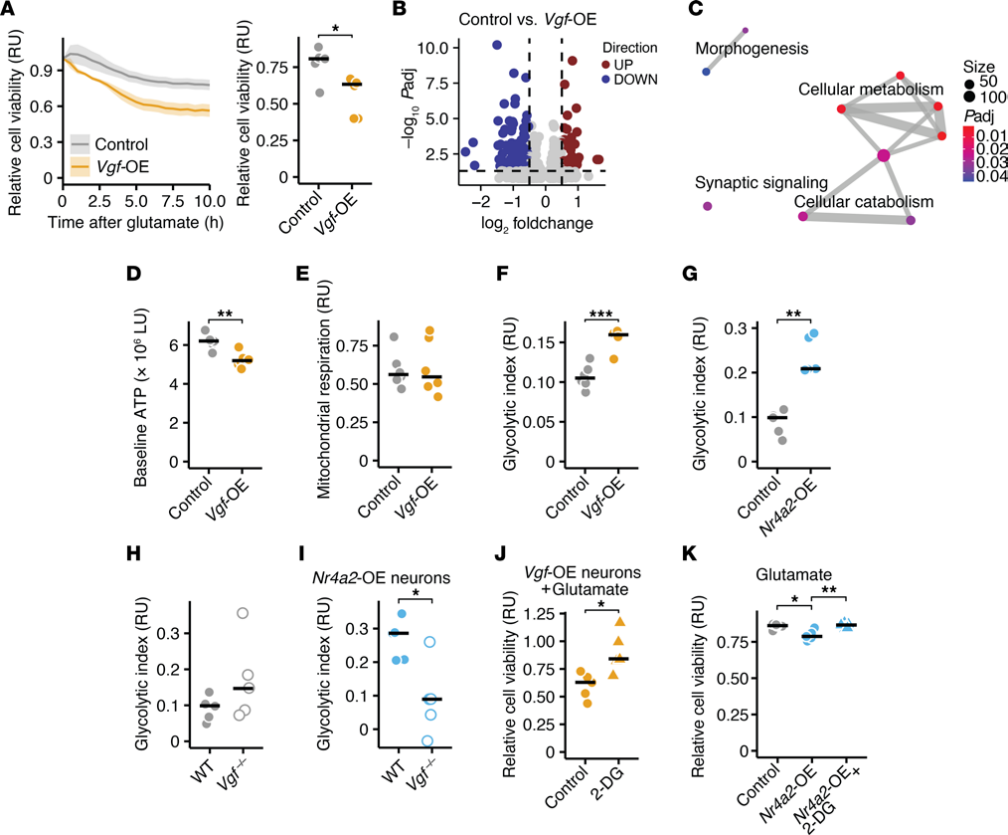
Persistent VGF exposure exacerbates excitotoxicity by inducing glycolysis. (**A**) Relative cell viability (RU, relative units) of neuronal cultures that overexpress EGFP (controls) or VGF and were exposed to glutamate (*n* = 6 per group). (**B**) Volcano plot showing all differentially expressed genes (DEGs) between control (EGFP) and VGF-overexpressing neurons. (**C**) GSEA of all DEG between control (EGFP) and VGF-overexpressing neurons. Size shows number of genes of GO terms, color shows significance. (**D**) Baseline ATP levels in control (EGFP) and VGF-overexpressing neurons (*n* = 6 per group). (**E** and **F**) Mitochondrial respiration (**E**) and glycolytic index (**F**) of control (EGFP) and VGF-overexpressing neurons (*n* = 6 per group). (**G**) Glycolytic index of control (mScarlet) and NR4A2-overexpressing neurons (*n* = 5 per group). (**H**) Glycolytic index of WT and *Vgf*-deficient neurons (*n* = 5 per group). (**I**) Glycolytic index of WT and *Vgf*-deficient neurons that overexpress NR4A2 (*n* = 5 per group). (**J**) Relative cell viability (RU) VGF-overexpressing neurons that were exposed to glutamate and were pretreated with vehicle (control) or 5 mM 2-DG (*n* = 5 per group). Data was normalized to controls without glutamate stimulation. (**K**) Relative cell viability (RU) of neuronal cultures that overexpress mScarlet (control) or NR4A2 and were exposed to glutamate and pretreated with vehicle or 5 mM 2-DG (*n* = 5 per group). Data was normalized to controls without glutamate stimulation. Points represent individual experiments, additionally, mean is shown. If not stated otherwise, unpaired *t* test with FDR correction for multiple comparisons was used. *P < 0.05, **P < 0.01, ***P < 0.001.

Given that chronic VGF expression was associated with neurodegeneration, we next explored its causality. By overexpressing VGF in neurons through AAV delivery, compared with EGFP controls, we detected no baseline differences in cell viability (Supplemental Figure 4A). However, we observed increased vulnerability to glutamate toxicity in VGF-overexpressing neuronal cultures (Figure 4A). This vulnerability was mitigated by changing the medium every other day, indicating that the secreted VGF is the main contributor to increased vulnerability. Similarly, applying the medium from VGF-overexpressing neurons to control neurons increased their vulnerability to glutamate excitotoxicity (Supplemental Figure 4B). To understand how chronic VGF exposure renders neurons more vulnerable to excitotoxicity, we sequenced the transcriptomes of VGF- and EGFP-overexpressing neuronal cultures (Figure 4B). GO term analyses revealed a range of differently regulated metabolic processes in VGF-overexpressing neurons (Figure 4C), while genes encoding other neurotrophic factors such as *Bdnf* and *Ngf* were not differentially regulated (Supplemental Figure 4C). Notably, well-established AD risk genes *Apoe*, *Cst3*, and *Clu* were significantly suppressed in VGF-overexpressing neurons (Supplemental Figure 4D).

Next, we assessed how VGF affects neuronal metabolism, as suggested by our sequencing data. To determine how neuronal metabolism is regulated by mitochondrial respiration and glycolysis, we measured ATP levels at baseline, after application of the respiratory chain inhibitors rotenone and oligomycin, and following the application of the glycolysis inhibitor 2-deoxy-D-glucose (2-DG) (35). Compared with EGFP-overexpressing neurons, chronic VGF expression reduced baseline ATP levels (Figure 4D), did not alter mitochondrial respiration (Figure 4E), and increased glycolytic respiration (Figure 4F). Since VGF is induced by NR4A2, we tested NR4A2-overexpressing neurons. Similar to VGF-overexpressing neurons, glycolytic respiration was increased in NR4A2-overexpressing neurons (Figure 4G), whereas baseline ATP levels and mitochondrial respiration (Supplemental Figure 4E) were unchanged compared with mScarlet-overexpressing control neurons. Of note, we did not observe differences in baseline ATP levels, mitochondrial respiration, or glycolytic respiration in NR4A1- (Supplemental Figure 4F) and NR4A3-overexpressing neurons (Supplemental Figure 4G). To determine whether increased glycolysis in NR4A2-overexpressing neurons was driven by VGF, we used *Vgf*-deficient neurons that were transduced with NR4A2 or mScarlet control expression constructs. mScarlet-overexpressing *Vgf*-deficient neurons showed no differences in baseline ATP levels, glycolytic index, or mitochondrial respiration (Figure 4H and Supplemental Figure 4H). Notably, the increase in glycolytic rate following NR4A2 overexpression was absent in *Vgf*-deficient neurons (Figure 4I). Since we observed increased vulnerability of VGF- and NR4A2-overexpressing neurons, we hypothesized that both observations converge into chronic activation of glycolysis. Treating mScarlet-, VGF-, and NR4A2-overexpressing neurons with the glycolysis inhibitor 2-DG followed by glutamate exposure mitigated the increased vulnerability to excitotoxic cell death observed in both VGF- and NR4A2-overexpressing neurons (Figure 4, J and K and Supplemental Figure 4I). We concluded that the NR4A2-VGF axis, which is chronically induced in an excitotoxic and neuroinflammatory environment, increases neuronal susceptibility by persistently activating glycolysis.

### Neuronal Vgf deficiency protects from neurodegeneration in EAE.

**Figure 5:**
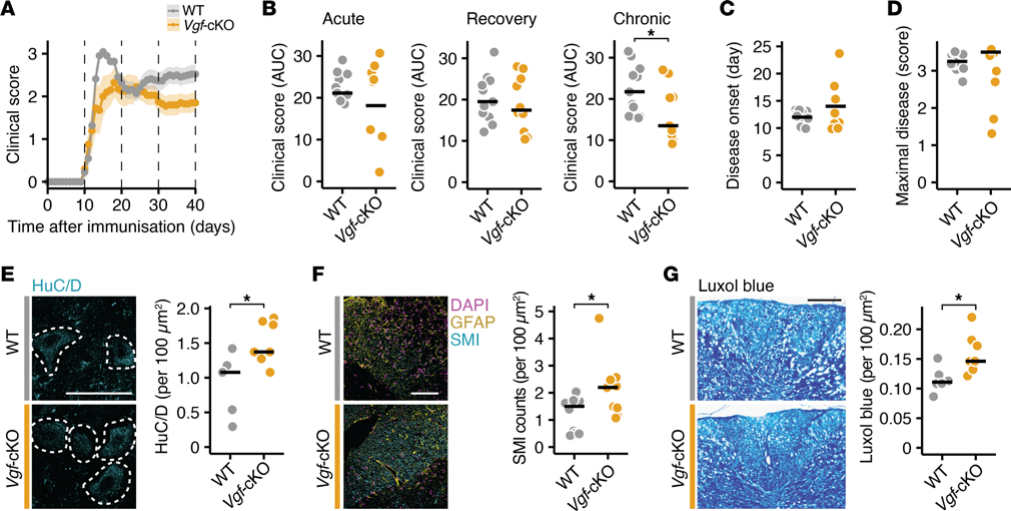
Neuronal VGF deficiency protects from neuroaxonal damage in EAE. (**A**) EAE disease course of *Snap25-Cre* × *Vgf^fl/fl^* (*Vgf*-cKO; *n* = 10) animals and respective *Vgf^fl/fl^* (*n* = 11) control littermates. Points represent mean per group per day, additionally, SEM is shown. (**B**) Cumulative clinical score using AUC during acute (days 11–20), recovery (days 21–30), and chronic (days 31–40) disease stages of EAE. (**C** and **D**) Disease onset (**C**) and maximal disease score (**D**) of *Vgf*-cKO and control mice. (**E**) Number of HuC/D-positive neurons in the ventral horn spinal cords of WT (*n* = 5) and *Vgf*-cKO (*n* = 7) EAE mice 40 days after immunization. Scale bar: 50 μm. (**F**) Number of axons in dorsal columns of the cervical spinal cord assessed by SMI staining in *Vgf*-cKO (*n* = 9) and control mice (*n* = 10) undergoing EAE 40 days after immunization. Scale bar: 50 μm. (**G**) Quantification of luxol fast blue-positive axonal area per 100 μm^2^ in the dorsal columns of the cervical spinal cords of WT (*n* = 6) and *Vgf*-cKO (*n* = 8) EAE mice 40 days after immunization. Scale bar: 500 μm. Points represent individual experiments, additionally mean is shown. For comparing EAE phenotypes nonparametric Kolmogorov-Smirnov test was used. For IHC analyses unpaired *t* test was used. *P < 0.05.

Finally, we tested whether our in vitro mechanistic findings can be translated to EAE. To selectively delete *Vgf* only in neurons, we crossed *Snap25*-Cre expressing mice with *Vgf*^fl/fl^ mice (*Vgf*-cKO) and induced EAE in these mice and their *Vgf*^fl/fl^ littermate control mice (Figure 5A). We observed an ameliorated disease course in *Vgf*-cKO during the chronic disease phase (Figure 5B and Supplemental Figure 5A). In contrast, the day of disease onset (Figure 5C) and the maximal disease score were similar (Figure 5D), suggesting that the immune response was unaffected. We recorded similar VGF levels in the blood of *Vgf*-cKO EAE mice (Supplemental Figure 5, B–E), whereas the CNS levels were significantly lower in *Vgf*-cKO EAE mice compared with WT EAE mice (Supplemental Figure 5F), suggesting that local but not systemic VGF secretion is driving neurological impairments in EAE mice. In line with the protective effect of neuronal *Vgf* deletion in the progressive disease stage, we observed an increased number of preserved neurons in the ventral horn of the spinal cord (Figure 5E) and axons in the dorsal columns of the cervical spinal cord in *Vgf*-cKO mice 40 days after immunization (Figure 5F). This was further confirmed by recording fewer damaged axons (Supplemental Figure 5G) and more myelinated axons (Figure 5G) in the dorsal columns of the cervical spinal cord in *Vgf*-cKO mice during chronic EAE. Glial scarring and microglial activation, estimated by quantifying glial fibrillary acid protein (GFAP) and the ionized calcium-binding adapter molecule 1 (IBA-1), respectively, were not different in *Vgf*-cKO mice (Supplemental Figure 5, H–J). Thus, chronic VGF secretion by inflammatory stressed neurons directly contributed to neurodegeneration in CNS inflammation.

## Discussion

We explored the impact of persistent neuronal hyperexcitability incited by CNS inflammation on neuronal vulnerability to cell death. Our research identified a continuously active NR4A2-VGF pathway that triggers neuronal demise, dependent on glycolysis, during CNS inflammation. NR4A2 was induced at both RNA and protein levels in neurons of *Grm8^–/–^* mice, which are more susceptible to inflammation-induced neurodegeneration due to glutamate-dependent hyperexcitability (25). We and others have previously shown that the biomarkers of neuronal excitation and glutamate signaling, pCREB (25) and its downstream transcription factor NR4A2 (26), are increased in neurons of pwMS. This is similar to postmortem findings in patients with epilepsy (36, 37) and, as we show here, in inflamed neurons during EAE, serving as histopathological biomarkers for a chronic hyperexcited state.

NR4A2 is highly expressed in the substantia nigra, where it serves as pivotal regulator of dopaminergic neuronal differentiation (38), explaining why familial PD syndromes are associated with *NR4A2* loss-of-function mutations (32). Additionally, NR4A2 is expressed in neurons throughout the entire CNS, and its temporary upscaling contributes to neurodevelopment, synaptic plasticity, and cognition (31, 39). Our findings suggest that chronic induction of NR4A2 is sensitizing neurons to cell death mechanisms during chronic CNS inflammation, which is induced by hyperexcitability. Notably, a protective role for NR4A2 has been demonstrated in an AD mouse model (40). However, in contrast to MS, NR4A2 is suppressed in neurons of patients with AD and PD (40, 41). Thus, chronic disequilibrium of NR4A2 induces neuronal demise, which can be prevented by rebalancing NR4A2 homeostasis. Additionally, our findings underscore that despite the numerous similarities of neurodegeneration between MS and neurodegenerative diseases like AD or PD, disease-specific mechanisms that determine neuronal cell death are at work. NR4A2 expression has also been described in glial cells and T cells where it exerts immunomodulatory functions. In line with this, we detected a NR4A2^hi^ nonneuronal population by nuclear flow cytometry. Notably, NR4A2 is upregulated in T cells of pwMS, and adoptive transfer EAE with *Nr4a2*-deficient T cells shows reduced disease severity, implying that NR4A2 acts as a proinflammatory regulator (42). Conversely, an antiinflammatory role for NR4A2 was described in macrophages and microglia (43, 44). Since immune cell activation is decisively regulated by the metabolic state (45), it is intriguing to speculate whether NR4A2 plays a similar role in immune cells as in neurons. Further studies are warranted to investigate whether NR4A2 modulation could be exploited to alter the immune landscape. Notably, we analyzed coexpression modules across 502 neuron-specific transcriptome data sets and did not find inflammatory genes to be coregulated with NR4A2. Instead, NR4A2 emerged as regulator of morphogenesis and neuronal growth during CNS inflammation.

The neuropeptide VGF was the top differentially regulated gene within the neuronal NR4A2-controlled network during CNS inflammation. Accordingly, we observed increased neuronal VGF in neurons overexpressing NR4A2, *Grm8*-deficient mice, and EAE mice. At the same time, we detected increased peripheral blood VGF levels in EAE mice. However, in our *Vgf*-cKO mice, these levels were not reduced compared with WT mice during EAE, in contrast to the reduced VGF levels in the CNS. Since active immunization in EAE mice induces tissue damage and a systemic inflammatory response, VGF may also be secreted by the neuroendocrine system, the peripheral nervous system (46, 47), and other organs (48) not targeted by our *Snap25*-Cre mouse line, potentially masking the blood levels in *Vgf*-cKO mice. Conversely, pwMS experience inflammation restricted to the CNS (4). Therefore, the elevated VGF levels in both the blood and in brain biopsies of pwMS likely reflect ongoing inflammatory challenges to neurons associated with an E/I imbalance. The chronic hyperexcitability might account for the cognitive deficits that are commonly observed in pwMS (49). The magnitude of cognitive dysfunction correlates with increased functional network connectivity measured by functional MRI (fMRI) in pwRRMS (50, 51). This increased connectivity is spatially correlated with chronic inflammatory activity in MRI (52), providing in vivo evidence that inflammation drives a hyperexcitable E/I state in pwMS. Mechanistically, the E/I shift in EAE is partially explained by TNF-α signaling (15) and a selective loss of inhibitory neurons during neuroinflammation (17). Additionally, in this study we demonstrated that the downregulation of the inhibitory GRM8 in motoneurons of EAE animals contributes to maintaining the hyperexcitable E/I state, which contrasts with our previous findings in postmortem brains of pwMS where GRM8 was expressed to a similar level as in controls (25). This discrepancy could be due to varying degrees of inflammation between EAE mice and progressive MS. Nevertheless, expression of GRM8 in lesions and nonlesioned grey matter in pwMS makes it an attractive treatment target. Further studies in pwMS are needed to systematically determine VGF expression in different organ systems during EAE and in pwMS and if blood levels of VGF or its different peptides correlate with network connectivity, which could promote VGF as biomarker for E/I disbalance.

Mechanistically, we discovered that the persistent VGF expression mediated the NR4A2-induced neuronal vulnerability. Chronic exposure to VGF rendered neurons more susceptible to excitotoxicity, while the neuronal deletion of VGF protected neurons from cell death in vitro and in EAE. This is surprising, as VGF has been identified as neuroprotective factor capable of slowing the disease course in an AD animal model (53). Notably, our transcriptome data set of VGF-overexpressing neurons showed significant downregulation of key AD pathophysiology players such as *Apoe*, *Cst3*, and *Clu* (54), indicating that VGF suppresses the transcription of AD-specific neurodegenerative drivers, which is not the case in MS. In addition, VGF is an important amplifier and facilitator of long-term potentiation and memory formation, activating the neurotrophic receptor tyrosine kinase 2 (also known as TRKB) in the hippocampus (55, 56). While hippocampal neurodegeneration is a hallmark of AD, pwMS experience more diffuse neurodegeneration. Notably, VGF CSF levels are chronically downregulated in patients with AD (57, 58) but elevated in pwMS, reflecting the divergent NR4A2 expression in the CNS of patients with AD and MS. In an AD mouse model, VGF inhibits proinflammatory microglia by binding to the complement C3a receptor 1 (C3aR1) (59, 60). In contrast, we did not find VGF-dependent differences of microglia numbers in EAE. Hence, our data strongly suggest that the NR4A2–VGF axis exerts distinct influences on neurodegeneration in both MS and AD. It is intriguing to speculate that the detrimental E/I imbalance in the early disease phase of MS (15) is unique to conditions where inflammation is the principal initiator of neuronal degeneration (61), unlike AD, where various neuron-intrinsic pathologies induce a reactive immune response to mitigate disease progression (62, 63). A systematic analysis of the differences between inflammation-induced neurodegeneration and neurodegeneration associated with secondary inflammation will facilitate a better differentiation between shared and unique hallmarks of neurodegeneration and disease-specific treatment targets and biomarkers.

In fact, NR4A2 and VGF were part of a highly upregulated module in motoneurons of EAE animals that regulates neuronal growth and morphogenesis. Similar regenerative transcriptional responses have been observed in mouse models of spinal cord injury (64, 65) and optic nerve crush, where VGF is similarly induced (66). Neurite and axonal growth require high energy demand and a continuous de novo synthesis of proteins and lipids (67). Although glia cells provide energetic support (68), neurons must increase glucose uptake and perform glycolysis with increasing excitation (69, 70). Proliferative neuronal progenitor cells similarly rely on glycolytic energy production (71). Concurrent with glycolysis, the pentose-phosphate pathway is activated, leading to an increased production of reactive oxygen species (72). Notably, we found that continuous NR4A2-VGF activation induced glycolysis, while inhibiting glycolysis counteracted the increased susceptibility to excitotoxicity. This effect was likely mediated by secreted VGF, as changing the medium rescued the phenotype of VGF-overexpressing neurons. Further studies should examine the role of intracellular VGF signaling, which regulates exocytosis in pancreatic islet cells and might similarly occur in neurons (73). Although deleting *Vgf* in neurons reversed the increase of NR4A2-dependent glycolysis, VGF overexpression increased the glycolytic index to a lesser extent than NR4A2 overexpression. Thus, VGF likely participates in an NR4A2-dependent secretory cascade that reprograms neuronal metabolism. Metabolic disturbances in CNS inflammation might be exacerbated by a reduction of oxidative phosphorylation and a depletion of tricarboxylic acid (TCA) cycle enzymes, as observed in EAE mice and pwMS (11). Since the TCA cycle metabolites tightly regulate the glycolytic rate (74), depletion of these enzymes could lead to disinhibition of glycolysis, as has been shown in other cell types (75). While the activation of the NR4A2-VGF axis might counteract acute challenges, its persistent activation appears to be a maladaptive response to chronic axonal damage, disruption of the TCA cycle, and oxidative phosphorylation in EAE and MS. Additionally, chronic exposure to cytokines that stimulate axonal growth during acute inflammation (72, 76) may further drive the long-lasting expression of NR4A2 and VGF.

In summary, we delineated how glutamate-mediated E/I imbalance drives neurodegeneration in CNS inflammation. Chronic induction of NR4A2-dependent neurotrophic VGF signaling causes glycolysis-dependent neuroaxonal damage in neuroinflammation. Thus, while neurotrophic factors are required for neuronal development, synaptic plasticity, and intercellular communication, their chronic secretion can elicit a maladaptive response in neurons during persistent CNS inflammation. This counterintuitive aspect must be considered when analyzing biomarkers and developing therapeutic strategies.

## Methods

### Sex as a biological variable.

Both male and female mice were examined. We did not observe sex-specific differences in any of the experiments; therefore, the sexes were reported together.

### Mice.

All mice (C57BL/6J WT [The Jackson Laboratory]; C57BL/6J *Grm8^–/–^*; C57BL/6J *Vgf^fl/fl^* (73); and C57BL/6J *Snap25-Cre* x *Vgf^fl/fl^*) were kept under specific pathogen-free conditions in the central animal facility of the University Medical Center Hamburg-Eppendorf (UKE). We used adult mice (6–20 weeks old) from both sexes; mice were sex- and age-matched in all experiments.

### EAE.

We immunized mice subcutaneously with 200 μg MOG35–55 peptide (Schafer-N) in CFA (Difco, DF0639-60-6) containing 4 mg/mL *Mycobacterium tuberculosis* (Difco, DF3114-33-8). In addition, we injected 200 ng pertussis toxin (Calbiochem, CAS70323-44-3) intraperitoneally on the day of immunization and 48 hours later. We scored animals daily for clinical signs by the following system: 0, no clinical deficits; 1, tail weakness; 2, hind limb paresis; 3, partial hind limb paralysis; 3.5, full hind limb paralysis; 4, full hind limb paralysis and fore limb paresis; 5, premorbid or dead. Animals reaching a clinical score at or above 4 were euthanized according to the regulations of the local Animal Welfare Act. The investigators were blind to the genotype and treatment in the EAE experiments.

### Primary neuronal cultures.

For primary cortical cultures we euthanized pregnant C57BL/6J, or *Vgf^fl/fl^* mice. We reserved tissue of each embryo for genotyping and isolated the cortex, dissociated, and plated cells at a density of 6 × 10^4^ cells per 1 cm^2^ on poly D-lysine-coated wells (5 μM Sigma-Aldrich). If not stated otherwise, cells were maintained in PNGM medium (Lonza) at 37°C, 5% CO_2_ and a relative humidity of 98%. Throughout, we used cultures after 14–23 days in vitro (div) for experiments. To generate *Vgf*-deficient neurons, neuronal cultures from homozygous *Vgf^fl/fl^* breedings were transfected at 7 div with an AAV7 containing a Cre expression plasmid (pENN.AAV.CMVs.Pl.Cre.rBG was a gift from James M. Wilson (Addgene plasmid 105537; http://n2t.net/addgene:105537; RRID:Addgene_105537)) with a MOI of 15,000. Cells were transduced with AAV7 harboring *Vgf* or a lentivirus containing *Nr4a2* at div 7 with a MOI of 15,000. For Supplemental Figure 4B, 80% of the medium of *Vgf*-overexpressing neurons was replaced with medium of mScarlet-overexpressing neurons and vice versa every other day. Where indicated, we treated neurons with 1 μM AZ12216052, 50 nM Ip7e, or 5 mM 2-deoxy-D-glucose every other day starting from 10 div.

### RealTime-Glo cell viability assay.

We mixed RealTime-Glo (Promega, G9711) MT cell viability substrate and NanoLuc Enzyme together, added it to neuronal cultures, and incubated them for 5 hours for equilibration of luminescence signal before respective treatments were applied. Toxicity was estimated after applying 50 μM glutamate. We recorded luminescence with a Spark 10 M multimode microplate reader (Tecan) at 37°C and 5% CO_2_ every 30 minutes over a total time of 15 hours after application of glutamate. We used at least 4 technical replicates per condition. For analysis, every well’s data point was normalized to its last value before the stressor was added and then normalized to the mean of the control wells for every time point. Thereby, we controlled for well-to-well seeding variability. The data points shown in Figure 1J and Figure 4K for glutamate stimulation of control and NR4A2-overexpressing neurons are identical, as the additional pharmacological treatments were applied in the same experiment. For statistical analysis we compared either AUC or end point.

### Vector construction.

To insert mouse *Vgf* into a pAAV, we first performed PCR using Primer_f_1 and Primer_r_1 from mouse cortex cDNA. Restriction and ligation were performed using KpnI and HindIII restriction sites. The reverse primer included a P2a domain that was inserted before an EGFP. We used a modified pAAV-hSyn-EGFP as backbone. pAAV-hSyn-EGFP was a gift from Bryan Roth (Addgene, 50465; http://n2t.net/addgene:50465; RRID: Addgene_50465). AAVs were produced according to the standard procedures of the UKE vector facility. *Nr4a1*, *Nr4a2*, and *Nr4a3* were inserted into a lentiviral backbone with a hSyn promoter ensuring neuronal expression and a P2a-mScarlet to visualize transduction efficacy. Nr4a1 was isolated using Primer_f_2 and Primer_r_2; Nr4a2 using Primer_f_3 and Primer_r_3; and Nr4a3 using Primer_f_4 and Primer_r_4. Restriction and ligation were performed using AgeI and XbaI restriction sites for *Nr4a2* and *Nr4a3*, and BsiWI and XbaI for *Nr4a1*. All final products were confirmed using Sanger sequencing. All primers used in this study are provided in Supplemental Table 5. For all AAV-delivered *Vgf* overexpression experiments, EGFP overexpression construct was used as control. For the lentiviral *Nr4a2* overexpression experiments, a lentiviral mScarlet overexpression construct was used as control.

### Lentiviral production.

To produce lentiviruses, we first transfected HEK293T cells with 10 μg expression plasmid, 10 μg pMDLg/pRRE, 5 μg pRSV-Re, and 2 μg pMD2.G. pMDLg/pRRE was a gift from Didier Trono (Addgene 12251; http://n2t.net/addgene:12251; RRID: Addgene_12251). pRSV-Rev was a gift from Didier Trono (Addgene 12253; http://n2t.net/addgene:12253; RRID: Addgene_12253). pMD2.G was a gift from Didier Trono (Addgene #12259; http://n2t.net/addgene:12259; RRID: Addgene_12259). Briefly, HEK293T cells were seeded out with an 80% confluency in DMEM with glutamine and high glucose (Thermo Fisher Scientific, 10569010), the next day the plasmids were mixed in 1 × HEPES buffered saline (HBS) and 125 mM CaCl_2_ and were applied to the HEK293T cells for 6 hours. Subsequently, medium was changed and after 48 hours the supernatant was filtered through a 0.45 μm PES filter and immediately snap frozen and stored at –80°C.

### Weighted gene correlated network analysis.

We performed weighted gene correlated network analysis (WGCNA) to identify coregulated genes of *Nr4a2* and *Vgf* using the WGCNA (77) package. We included in total 502 publicly available samples from neurons under steady-state or different in vitro or in vivo stress models. All included data sets are provided in Supplemental Table 1. Modules were constructed by unsigned Pearson’s correlation using a soft threshold power of 10 corresponding to a scale free model fit greater-than 0.9. In total, 93 modules were found. We excluded 13 modules with less than 5 genes and more than 1,000 genes. Genes were assigned to a module when the adjusted module membership *P* value less-than 0.05. To test the enrichment of inflamed motoneuron signatures during EAE, we performed gene set enrichment analysis (GSEA) with a ranked gene list obtained from GSE104899 using the clusterProfiler (78) package. To construct the motoneuron-specific signature, we filtered for genes that were only differentially regulated in motoneurons but not in the whole spinal cord of EAE mice and genes that were higher expressed in motoneurons than in the whole spinal cord, as we have done previously (9). To calculate the association between *Vgf* expression and GO terms, we performed biological process GO term enrichment analysis with each module. Next, we performed Pearson correlation analysis with *Vgf* module modularity and GO term NES.

### RNA-Seq and analysis.

Primary neurons were transduced at 7 div with AAV7 containing *Vgf* or EGFP with a MOI of 15,000 and harvested at 16 div. Until further processing, samples were stored at –80 °C. Total RNA was isolated from the fresh frozen samples using RNeasy mini kit (Qiagen, 74106), following the manufacturer’s suggested protocol. RNA sequencing libraries were prepared using the TruSeq stranded mRNA Library Prep Kit (Illumina) according to the manufacturer’s manual (document 1000000040498 v00) with a minimum total RNA input of 100 ng per sample. Libraries were pooled and sequenced on a NovaSeq 6000 sequencer (Illumina) generating 50 bp paired end reads. The reads were aligned to the Ensembl mouse reference genome (GRCh39) using STAR v.2.4 (79) with default parameters. The overlap with annotated gene loci was counted with featureCounts v.1.5.1 (80). Differential expression analysis was performed with DESeq2 (v.3.12) (81) calling genes with a minimal 2-fold change and FDR-adjusted *P* < 0.05 differentially expressed. Gene lists were annotated using biomaRt (v.4.0). GSEA was performed using the clusterProfiler (78) package.

### Real-time PCR.

We reverse transcribed RNA to complementary DNA with the RevertAid H Minus First Strand cDNA Synthesis Kit (Thermo Fisher Scientific) according to the manufacturer’s instructions. We analyzed gene expression by real-time PCR performed in an ABI Prism 7900 HT Fast Real-Time PCR System (Applied Biosystems) using the TaqMan Gene Expression Assays (Thermo Fisher Scientific) for *Nr4a2* (Mm00443060).

### Patient cohorts.

pwMS and healthy individuals were recruited through the MS outpatient clinic of the Department of Neurology, University Medical Center Hamburg-Eppendorf (UKE). The study was approved by the local ethics committee (Hamburg Chamber of Commerce Act for the Health Professions, registration number PV4405). Informed consent was obtained from all patients and nonneurological disease controls. The sera were acquired and processed according to the standard operating procedures of the UKE, Institute of Neuroimmunology and Multiple Sclerosis (INIMS). All cohorts from which sera were analyzed were compiled from cryopreserved samples collected in the biobank of the INIMS where the samples are stored at –80°C. The patients’ characteristics are shown in Supplemental Table 3.

### VGF ELISA.

Competitive enzyme-linked immunosorbent assay *(*ELISA) was set with antisera raised against synthetic VGF peptides, as shown in the Supplemental Table 6. Multiwell plates (Nunc) were coated with the relevant synthetic peptides and treated with PBS (containing 9% normal serum from the secondary antibody donor species, 0.2 mg/mL sodium azide, and 1 mg/mL EDTA) for 2 hours. Primary incubations with VGF antibodies were carried out in triplicates, including serial standard dilutions in parallel with samples (final dilution 1:20). Biotinylated secondary antibodies (Jackson), streptavidin-peroxidase conjugate (Biospa), and tetrametylbenzidine (TMB X-traKem-En-Tec) as substrate were used to reveal the positive labelling. Hence, the reaction was stopped with HCl (1 mol/L) and the optical density was measured at 450 nm using a multilabel plate reader Tecan Spark 10M. To estimate whole VGF levels, we built z-scores of each measured peptide that were averaged and normalized to the respective control cohorts (see above). One biological GGGE measurement in acute EAE was removed due to technical issues where no signal was detected.

### Supplemental Methods.

Further information can be found in Supplemental Methods.

### Statistics.

The statistical analyses applied during the bioinformatics analysis are detailed in the respective sections of the article. Flow cytometric data were analyzed by using FlowJo (LLC). Images were analyzed using Fiji software (NIH). Experimental data were analyzed within the R environment on a Mac OS. Unless stated otherwise, the data are presented as means, and differences between 2 experimental groups were determined using unpaired, 2-tailed Student’s *t* tests and were FDR corrected for multiple comparisons. Statistical analysis of the clinical scores in the EAE experiments was performed by applying a Kolmogorov-Smirnov statistical test to the AUCs for each animal during the acute (10 days to 20 days post immunization [p.i.]), recovery (21 days to 30 days p.i.), and chronic (31 days to 40 days p.i.) disease phases. Correlation analyses were performed using Pearson-correlation. *P* values less than 0.05 were considered significant.

### Study approval.

All animal care and experimental procedures were performed according to institutional guidelines and conformed to the requirements of the German Animal Welfare Act. Ethical approvals were obtained from the State Authority of Hamburg, Germany (approval no. 41/22). Ethical approval for drawing and analyzing blood samples from pwMS and healthy controls were obtained from the Hamburg Chamber of Commerce Act for the Health Professions (approval no. PV4405). As human tissue could no longer be assigned to a human being, the analyses did not constitute a “research project on humans” in the sense of § 9 para. 2 of the Hamburg Chamber of Commerce Act for the Health Professions and therefore did not require consultation in accordance with § 15 para. 1 of the Professional Code of Conduct for Physicians in Hamburg.

### Data availability.

Data generated for this study are available through the Gene Expression Omnibus under accession number GSE247078. The data sets used for WGCNA analysis are listed in Supplemental Table 1. The signatures from inflamed motoneurons in EAE were obtained from GSE104899. Values for data shown in graphs can be found in the Supporting Data Values file.

### Code availability.

No original source code was generated in this study. The scripts for statistical analyses are available from the corresponding author upon reasonable request.

## Author contributions

MSW, LCB, and MAF designed the study. MSW and LCB performed most experiments. MSW performed bioinformatical analyses. I Winschel, NM, MG helped with biobanking and human cohorts. BS, I Winschel, MW, CM, LBL, NR, LU, LR, VV, JKS, and AH helped with mouse experiments. EM, BN, ALM, and CC performed ELISA experiments. OP helped with ChIP. SBS provided mice, reagents, and expertise. GDL, I Wagner, and DM performed RNAscope experiments and analyses. MSW, LCB, and MAF wrote the initial version of the manuscript. MSW and MAF supervised the study. MAF funded the study. All coauthors contributed to the editing and discussion of the manuscript and approved the final version.

## Supplementary Material

Supplemental data

Unedited blot and gel images

Supplemental table 1

Supplemental table 2

Supporting data values

## Version 1 08/15/2024

Electronic publication
