# Supplementary material for: The NR4A2/VGF pathway fuels inflammation-induced neurodegeneration via promoting neuronal glycolysis
Source: J Clin Invest. 2024 Jun 18;134(16):e177692. doi: 10.1172/JCI177692 (PMC11324305; doi:10.1172/JCI177692)
Supplement: Supplemental data [file jci-134-177692-s022.pdf]

SUPPLEMENTAL MATERIAL

The NR4A2/VGF pathway fuels inflammation-induced neurodegeneration via promoting neuronal glycolysis

Marcel S. Woo[1,*], Lukas C. Bal[1,*], Ingo Winschel[1], Elias Manca[1,2], Mark Walkenhorst[1], Bachar Sevgili[1], Jana K. Sonner[1], Giovanni Di Liberto[3], Christina Mayer[1], Lars Binkle-Ladisch[1], Nicola Rothammer[1], Lisa Unger[1], Lukas Raich[1], Alexandros Hadjilaou[1,4], Barbara Noli[2], Antonio L. Manai[2], Vanessa Vieira[1], Nina Meurs[1], Ingrid Wagner[3], Ole Pless[5], Cristina Cocco[2], Samuel B. Stephens[6], Markus Glatzel[7], Doron Merkler[3], Manuel A. Friese[1,§]

[1]Institute of Neuroimmunology and Multiple Sclerosis, University Medical Center Hamburg-Eppendorf, Hamburg, Germany
[2]Department of Biomedical Sciences, NEF-Laboratory, University of Cagliari, Monserrato, Cagliari, Italy
[3]Department of Pathology and Immunology, Division of Clinical Pathology, Faculty of Medicine, University of Geneva, Geneva, Switzerland
[4]Protozoa Immunology, Bernhard-Nocht-Institute for Tropical Medicine (BNITM), Hamburg, Germany.
[5]Fraunhofer Institute for Translational Medicine and Pharmacology ITMP, Hamburg, Germany
[6]Department of Internal Medicine, Fraternal Order of Eagles Diabetes Research Center, University of Iowa, Iowa City, US
[7]Institute of Neuropathology, University Medical Center Hamburg-Eppendorf, Hamburg, Germany
*These authors contributed equally.
§Corresponding author

SUPPLEMENTAL METHODS

Calcium imaging. We seeded primary neuronal cultures on either Ibidi 60 µ-Dish Quad (cat. 80411) or High (cat. 81158) with a glass bottom. To measure cytosolic calcium changes, we transduced neuronal cultures with an AAV7 containing pAAV-Syn-GCamp6f-WPRE-SV4088 (Addgene 100837) at 8–12 d.i.v. with a 10,000–20,000 MOI. AAV particles were produced according to the standard procedures of the UKE vector facility. We acquired images with a confocal LSM 700 laser scanning confocal microscope (Zeiss) every 0.48 seconds with a 20× magnification in an imaging chamber maintaining 37 °C and 5% CO_2. Infected cultures were imaged in the respective culture medium. In general, we recorded the first 5–10 minutes of baseline activity before applying the indicated chemicals. At the end of recording, we applied 10 µM ionomycin to induce maximum cellular calcium response that was used for normalization. Specific assay details and concentrations can be found in the respective figure legend. For data analysis, we measured mean fluorescence values of every cell using Fiji software (NIH) and normalized it to the maximal calcium response after ionomycin challenge (indicated as F/F_{Max}). For each cell, we calculated maximal, minimal, mean and AUC of the calcium response using a custom R script. If not stated otherwise, AUC was used for statistical comparisons.

Flow cytometric nucleus sorting. Nuclei of mouse spinal cords were isolated with the Nuclei Isolation Kit (Sigma-Aldrich, cat. NUC101) according to the manufacturer's protocol. To obtain neuronal nuclei, we stained nuclei with propidium iodide (1:2000; BioLegend; cat. 421301) and a primary labelled antibody directed against NeuN (1:500; abcam, ab190565). Then we sorted $PI^{+}NeuN^{+}$ nuclei by using a BDAriaIII cell sorter (BD Bioscience). We processed RNA for real-time PCR as described above. For flow cytometric analysis of NR4A2 intensity in cortical neurons, we fixed nuclei by methanol and subsequently stained them with antibodies directed against NR4A2 (1:500; Merck, ABE1455) and NeuN (1:500; abcam, cat. ab190565), as well as Hoechst33342 (1:500; ThermoFisher, cat. 62249) and the respective secondary antibodies, donkey anti-rabbit Alexa Fluor 488 (1:500, abcam, cat. 1:500; abcam, ab150105) and goat anti-chicken Alexa Fluor 647 (1:500; abcam, cat. Ab97145). We analyzed stained nuclei using a BD LSR II flow cytometer (BD Biosciences).

RNAscope *in situ* hybridization. We performed RNAscope fluorescent *in situ* hybridization (FISH) using the RNAscope Fluorescent Multiplex Kit V2 (Advanced Cell Diagnostics, cat. 323100) according to the manufacturer's protocol. Probes against murine Mm-*Snap25* (cat. 516471) and Mm-*Grm8*-C2 (cat. 521491-C2) were commercially available from Advanced Cell Diagnostics, Inc. RNAscope. We acquired single-plane confocal images with Zeiss LSM800 microscope, sampling spinal cord tissue at 63× magnification. We automatically stitched acquired tiles using ZEN imaging software (Zeiss). For each mouse Snap25+ neurons (*n* = 200–388) were evaluated for Grm8 signal. $Grm8^{+}Snap25^{+}$ neurons were quantified in the ventral horns of cervical spinal cords by a blinded experimenter using Pannoramic Viewer software (3DHISTECH) and Fiji (NIH Image analysis), or with a custom-made script, which was based on Cognition Network Language (Definiens Cognition Network Technology; Definiens Developer XD software).

Immunocytochemistry. For immunocytochemistry of mouse neurons, we cultivated cultures on 12 mm diameter coverslips. They were stimulated with 50 µM glutamate or when indicated additionally with 50 µM 2-APB and 2 mM EDTA for 6 hours. Subsequently, they were fixed with 4% paraformaldehyde (PFA) and incubated in 10% normal donkey´s serum (NDS) containing 0.1% Triton X-100 and subsequently immunolabeling was performed. To visualize neuronal morphology, we used acti-stain 555 phalloidin (1:100, cat. PHDH1-A, Cytoskeleton), actin-stain 670 phalloidin (1:100, cat. PHDN1-A, Cytoskeleton). Furthermore, we measured VGF (Santa Cruz; 1:100, cat. sc-365397) and NR4A2 (1:500; Merck, cat. ABE1455). Images were acquired using a confocal LSM 700 laser scanning confocal microscope (Zeiss).

Mouse histopathology. Mouse spinal cord tissue and cortex tissues was obtained and processed as described previously (1). Axons and myelination in EAE mice were quantified in transverse sections of the dorsal columns in the cervical spinal cord. The number of neurons and microglia morphology were quantified in the ventral horn of the cervical spinal cord. Mouse sections were analyzed with a Zeiss LSM 700 confocal microscope. We stained VGF (Santa Cruz; 1:100, cat. sc-365397), neurofilament (BioLegend; 1:300, cat. 837904), GFAP (Millipore; 1:500, cat. AB5541), IBA-1 (Synaptic Systems, 1:500, cat. 234308), HuC/D (ThermoFisher; 1:100, cat. A21271), and NFL DegenoTag (EnCor Biotecnology; 1:100, cat. CPCA-NF-L-Degen). Images were acquired using a confocal LSM 700 laser scanning confocal microscope (Zeiss) or LSM 900 Airyscan 2 confocal microscope (Zeiss). For 3,3'-diaminobenzidine (DAB) staining of NR4A2, we used hematoxylin (blue color) and immunolabeling, that we visualized using the avidin–biotin complex technique with 3,3′-diaminobenzidine (brown stain). We analyzed slides with a NanoZoomer 2.0-RS digital slide scanner and NDP.view2 software (Hamamatsu). For luxol fast blue staining, we quantified the area of luxol blue-positive axons in the white matter of the dorsal columns in the cervical spinal cord using a customized counting mask with Fiji (ImageJ). This was normalized to the total analyzed areas in the dorsal columns in the cervical spinal cords for quantification.

Human histopathology. The specimen used for histopathology were obtained from brain biopsies of pwMS, where MS-typical changes were described by board-certified histopathologists, and the patients had a MS diagnosis as determined by board-certified neurologists. The specimens were anonymized (cohort characteristics are provided in **Supplemental Table 4**). The use of patient specimens for research upon anonymization was in accordance with local ethical standards and regulations at the University Medical Center Hamburg-Eppendorf. We deparaffinized the sections using a standard ethanol/xylol dilution. After rinsing the deparaffinized sections in water we performed antigen retrieval using a sodium citrate buffer (pH = 6.5). After permeabilization with 0.1% Triton and blocking with 10% NDS immunolabeling was performed with antibodies against NeuN (1:200; Millipore, ABN91), VGF (Santa Cruz; 1:100, cat. sc-365397), and DAPI. Images were acquired using a confocal LSM 700 laser scanning confocal microscope (Zeiss).

Estimation of mitochondrial and glycolytic respiration. We used an assay adapted from (2) to measure mitochondrial and glycolytic respiration. We measured ATP levels using the CellTiter-Glo Luminescent Cell Viability Assay (Promega, cat. G7570) which was performed according to

manufacturer's protocol. We recorded luminescence with a Spark 10M multimode microplate reader (Tecan). Per plate we measured ATP levels in neurons without stimulation, stimulation with 10 µM oligomycin and 0.5 µM rotenone, or stimulation with 10 µM oligomycin and 0.5 µM rotenone and 5 mM 2-DG. The mitochondrial respiration was defined as difference between baseline ATP and ATP levels after respiratory chain blockade. The glycolytic respiration was defined as difference between ATP levels after respiratory chain blockade and ATP levels after respiratory chain and glycolysis blockade. At least four technical replicates were used per condition which were averaged per biological replicate for statistical analysis.

Immunoblot. Spinal cords of healthy and EAE mice were homogenized using a tissue grinder in 2 mL radioimmuno-precipitation buffer (50 mM Tris, 150 mM NaCl, 0.5 mM EDTA, 10% SDS, 1% NP-40, 10% sodium deoxycholate, protease and phosphate inhibitor cocktails (cOmplete, Roche)), incubated at 4 °C for 30 minutes on a rotating wheel, and centrifuged for 5 minutes to remove the cell debris. After measuring the protein concentrations by a BCA assay (Pierce BCA Protein Assay Kit, ThermoFisher) according to the manufacturer's protocol, we used 25 µg protein for subsequent loading on SDS-pages (NuPAGE, ThermoFisher) and wet transfer on polyvinylfluoride membranes. Blocking was performed using 5% BSA for 1 hour at room temperature. First antibodies were incubated overnight at 4 °C. We quantified VGF (Santa Cruz; 1:500, cat. sc-365397) and NR4A2 (Merck; 1:500, cat. ABE1455) and used tubulin as normalization control (Merck; 1:1,000, cat. T8578). Horseradish peroxidase-labeled secondary antibodies (LI-COR, 1:20,000, cat. 926-80011) were applied for 1 hour at room temperature and chemiluminescence was visualized using WetsernSure PREMIUM Chemiluminescent Substrate (LI-COR) according to the manufacturer's protocol.

Chromatin immunoprecipitation (ChIP). ChIP was performed using the SimpleChIP Enzymatic Chromatin IP Kit (Cell Signaling, cat. 9003) according to the manufacturer's protocol with modifications. All buffers were prepared according to the manufacturer's protocol. We sacrificed three mice that were immediately perfused with 10 mL ice-cold PBS and the cortices were removed and stored on ice. The tissue was chopped to approximately 1 mm sized pieces and incubated with 1% formaldehyde and proteinase inhibitor cocktail (PIC) for 10 minutes on a thermomixer at 400 rpm. The cross-linking was stopped by adding glycine. Subsequently, the cross-linked tissue was washed twice with ice-cold PBS and PIC (centrifugation 1500 × g, 5 minutes, 4 °C) before being disintegrated into a single-cell suspension by 20 strokes of a dounce homogenizer. The nuclei were isolated by centrifugation with 1500 × g at 4 °C. The pellet was resuspended in buffer A (containing dithiothreitol and PIC) and was constantly inverted while being incubated for 10 minutes on ice. Subsequently, the suspension was centrifuged at 3000 × g for 5 minutes before 2 wash steps with buffer B (containing dithiothreitol and PIC). Subsequently, 0.5 µL micrococcal nuclease was added to the cell suspension and incubated for 20 minutes at 37 °C on a thermomixer shaking with 200 rpm until the digest was terminated by adding 10 µL 0.5 M EDTA. Subsequently, the nuclei were pelleted by centrifuging with 13,000 rpm for minute at 4°C. The pellet was resuspended in 100 µL ChIP buffer and incubated for 10 minutes on ice. Subsequently, the sample was sonicated using the Bioruptor 2000 (intensity: H, interval: 0.5, time: 8 minutes) before clarifying the lysate by centrifugation with 10,000 rpm for 10 minutes at 4 °C. For immunoprecipitation we added 5 µL antibody against NR4A2 (Merck; 1:500, cat. ABE1455)

and as negative control 2 µg of the provided IgG and incubated the samples rotating overnight at 4 °C. Subsequently, the immunoprecipitated proteins were isolated using magnetic protein G beads according to the manufacturer's protocol. To remove the beads, the final pellets were solved in 150 µL ChIP elution buffer and incubated for 30 minutes on 65 °C on a thermomixer shaking with 400 rpm. Using a magnetic rack, the supernatant was removed and 6 µL 5 M NaCl and 2 µL proteinase K were added before incubating the sample for 2 hours at 65 °C to reverse the cross-linking. The remaining lysate was used to isolate DNA according to the manufacturer's protocol. We designed primers that amplified the flanking 125 bp of the consensus nuclear receptor binding motif AGGTCA (3) 1757 base pairs upstream of the transcription starting site of *Vgf* using the following primers: fwd – TACAGAATTTGCTGGAGATGGAATCC, rev – TTCCCCATGGACAAGAAGTGAAACA. Additionally, we performed PCR with primers that amplify a 125 bp segment in the 3' untranslated region of *Vgf* 157 bp downstream of the stop codon where NR4A2 binding is not expected, using the following primers: fwd – ACAAGGCCCCGCCTCGGG, rev – TCGCTAGAGAAACTAGATGGGGAC. We performed PCR from the respective immunoprecipitated DNA and a 5% input control.

References

1. Schattling B et al. TRPM4 cation channel mediates axonal and neuronal degeneration in experimental autoimmune encephalomyelitis and multiple sclerosis. *Nat. Med.* 2012;18(12):1805–1811.
2. Stephens SB et al. The Prohormone VGF Regulates β Cell Function via Insulin Secretory Granule Biogenesis. *Cell Rep.* 2017;20(10):2480–2489.
3. Jiang L et al. Structural basis of binding of homodimers of the nuclear receptor NR4A2 to selective Nur-responsive DNA elements. *J. Biol. Chem.* 2019;294(51):19795–19803.
4. Schattling B et al. Bassoon proteinopathy drives neurodegeneration in multiple sclerosis. *Nat. Neurosci.* 2019;22(6):887–896.
5. Stark M, Danielsson O, Griffiths WJ, Jörnvall H, Johansson J. Peptide repertoire of human cerebrospinal fluid: novel proteolytic fragments of neuroendocrine proteins. *J. Chromatogr. B Biomed. Sci. Appl.* 2001;754(2):357–367.
6. Brancia C et al. VGF Protein and Its C-Terminal Derived Peptides in Amyotrophic Lateral Sclerosis: Human and Animal Model Studies. *PLoS One* 2016;11(10):e0164689.
7. van Steenoven I et al. Identification of novel cerebrospinal fluid biomarker candidates for dementia with Lewy bodies: a proteomic approach. *Mol. Neurodegener.* 2020;15(1):36.
8. Bernay B et al. Discovering New Bioactive Neuropeptides in the Striatum Secretome Using in Vivo Microdialysis and Versatile Proteomics. *Mol. Cell. Proteomics* 2009;8(5):946–958.
9. Sasaki K, Takahashi N, Satoh M, Yamasaki M, Minamino N. A Peptidomics Strategy for Discovering Endogenous Bioactive Peptides. *J. Proteome Res.* 2010;9(10):5047–5052.
10. D'Amato F et al. VGF Peptide Profiles in Type 2 Diabetic Patients' Plasma and in Obese Mice. *PLoS One* 2015;10(11):e0142333.

SUPPLEMENTAL FIGURES

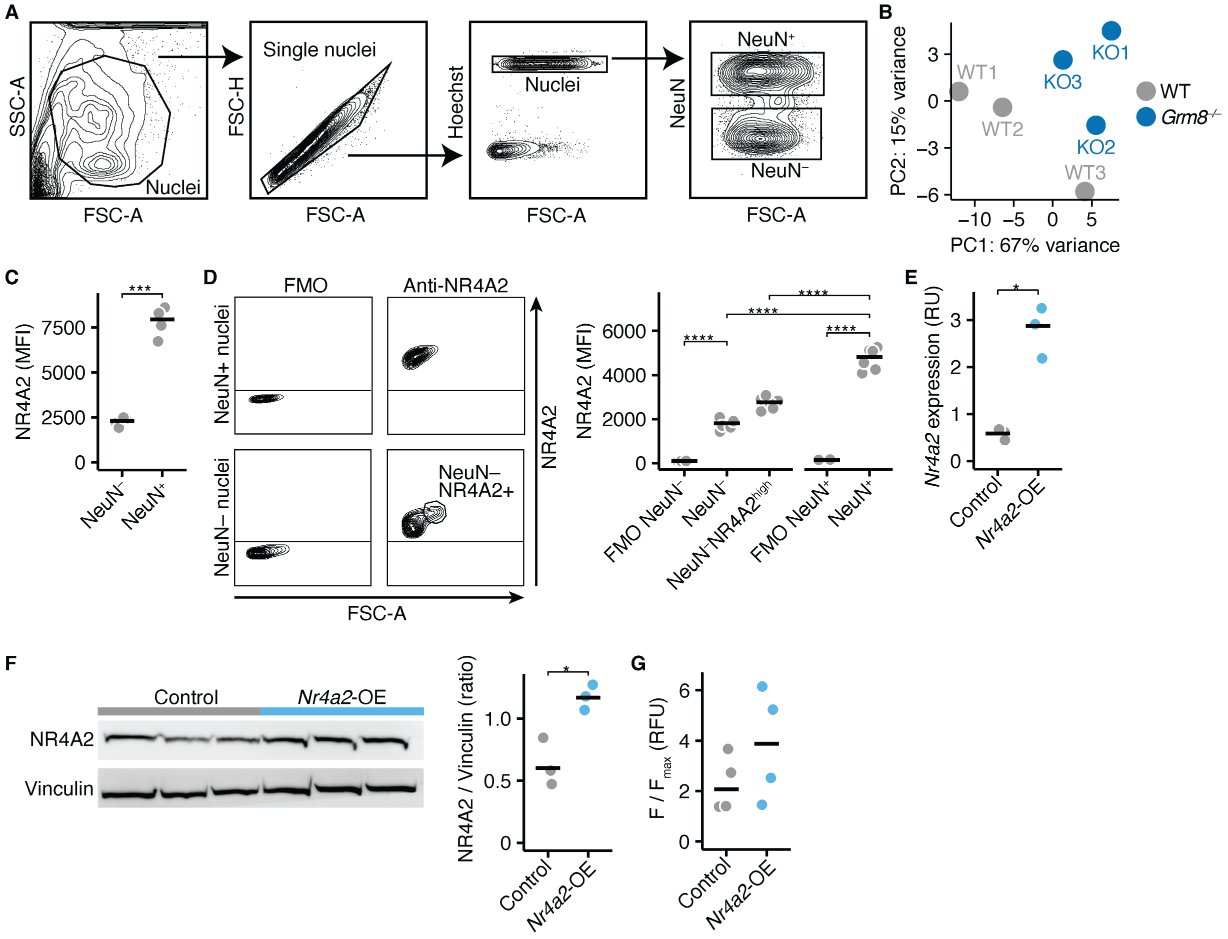

Supplemental Figure 1. NR4A2 is predominantly expressed in neurons in the CNS. (**A**) Representative gating scheme to isolate NeuN^{+} nuclei by flow cytometry. (**B**) Principal component analysis of our RNA-sequencing results from cortical neuronal nuclei of WT and *Grm8*$^{-/-}$ mice (*n* = 3 per group). (**C**) NR4A2 MFI in NeuN^{-} and NeuN^{+} cortical nuclei (*n* = 4 per group). (**D**) NR4A2 MFI in NeuN^{-}, NeuN^{-}NR4A2^{high}, and NeuN^{+} cortical nuclei. Additionally, fluorescence minus one control (FMO) where the primary antibody against NR4A2 was not added were measured for NeuN^{-} and NeuN^{+} nuclei (*n* = 6 per group). (**E**) *Nr4a2* mRNA expression of neuronal cultures that overexpress mScarlet (control) or *Nr4a2* (*n* = 3 per group) in relative units (RU). (**F**) Immunoblot analysis of NR4A2 protein levels in neuronal cultures that overexpress mScarlet (control) or *Nr4a2* (*n* = 3 per group). (**G**) Cumulative cytosolic calcium levels in mScarlet and *Nr4a2* overexpressing neurons (*n* = 4). Points represent individual experiments, additionally mean is shown. If not stated otherwise, unpaired *t*-test with FDR-correction for multiple comparisons was used. *P < 0.05, ***P<0.001, ****P < 0.0001.

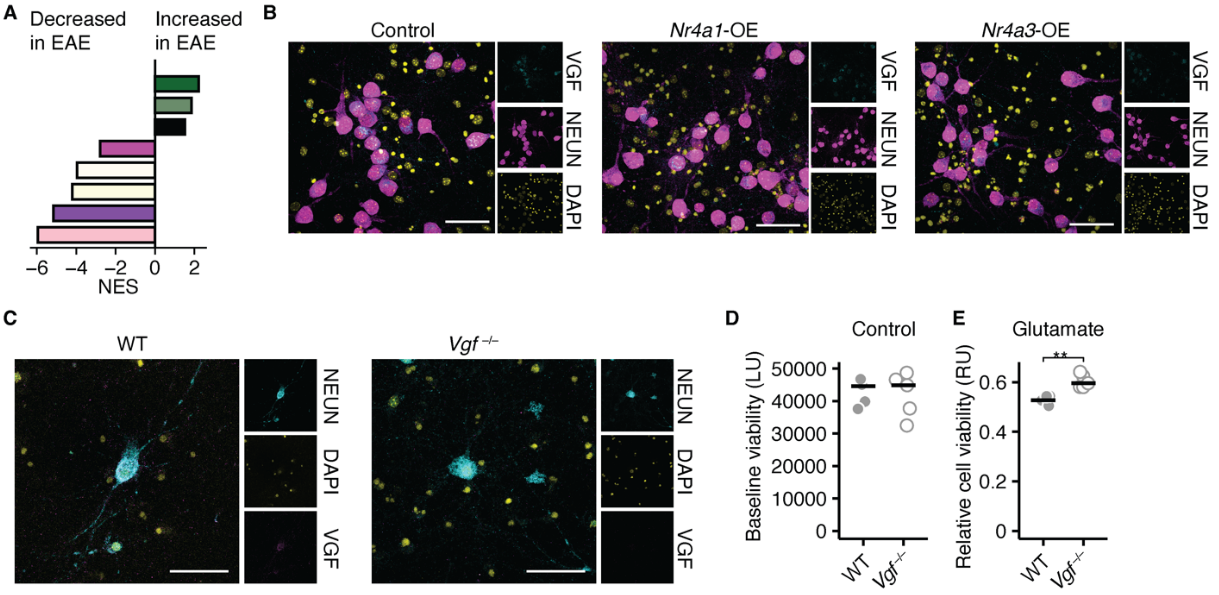

Supplemental Figure 2. VGF deficiency protects from glutamate excitotoxicity. (**A**) Enrichment analysis of inflamed neuronal signature (ranked gene list retrieved from (4)) in identified modules that contain *Nr4a2*. The normalized enrichment score (NES) is shown. All *P* values for enrichment for depicted modules < 0.05. (**B**) Representative images of mScarlet (controls), NR4A1-, and NR4A3-overexpressing neurons. Shown are VGF, NeuN and DAPI. Scale bar shows 20 µm. (**C**) Representative images of WT and *Vgf*$^{-/-}$ neurons. Scale bar shows 30 µm. (**D**) Baseline cell viability of WT and *Vgf*-deficient neurons (n = 5 per group). (**E**) Relative cell viability of WT and *Vgf*-deficient neurons after glutamate exposure (n = 5 per group) in relative units (RU). Points represent individual experiments, additionally mean is shown. If not stated otherwise, unpaired *t*-test with FDR-correction for multiple comparisons was used. ***P* < 0.01.

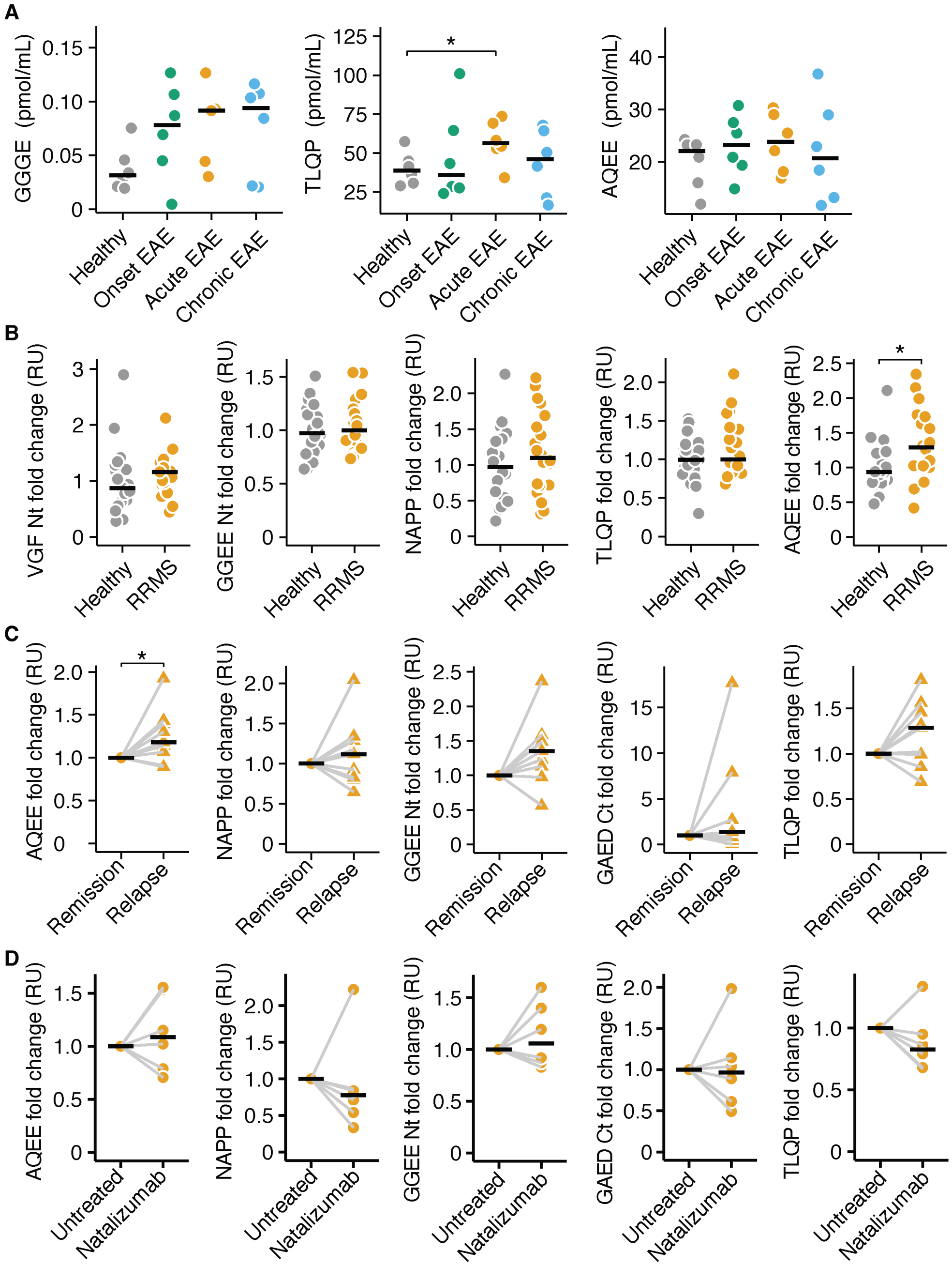

Supplemental Figure 3. VGF peptides in the plasma of EAE animals and pwMS. (**A**) Absolute levels of VGF peptides GGGE Nt (pmol/mL), TLQP (pmol/mL), and AQEE (pmol/mL) in plasma of EAE healthy mice and EAE animals 10 days (onset), 15 days (acute), and 30 days (chronic) after immunization (n = 6 per group; only GGGE acute n = 5). (**B**) Relative fold change of VGF peptides N-terminus of VGF, GGEE N-terminus, NAPP, TLQP, and AQEE in sera of healthy individuals and pwRRMS (n = 20 per group). (**C**) Relative fold change of VGF peptides AQEE, NAPP, TLQP, and GGEE N-terminus, GAED C-terminus, TLQP, in sera of pwRRMS during stable disease and relapse (n = 9 per group). Paired *t*-test was used for statistical comparisons. (**D**) Relative fold change of VGF peptides AQEE, NAPP, TLQP, and GGEE N-terminus, GAED C-terminus, TLQP, in sera of pwRRMS before and after treatment with natalizumab (n = 6 per group). Paired *t*-test was used for statistical comparisons. Points represent individual experiments, additionally mean is shown. If not stated otherwise, unpaired *t*-test with FDR-correction for multiple comparisons was used. *P < 0.05.

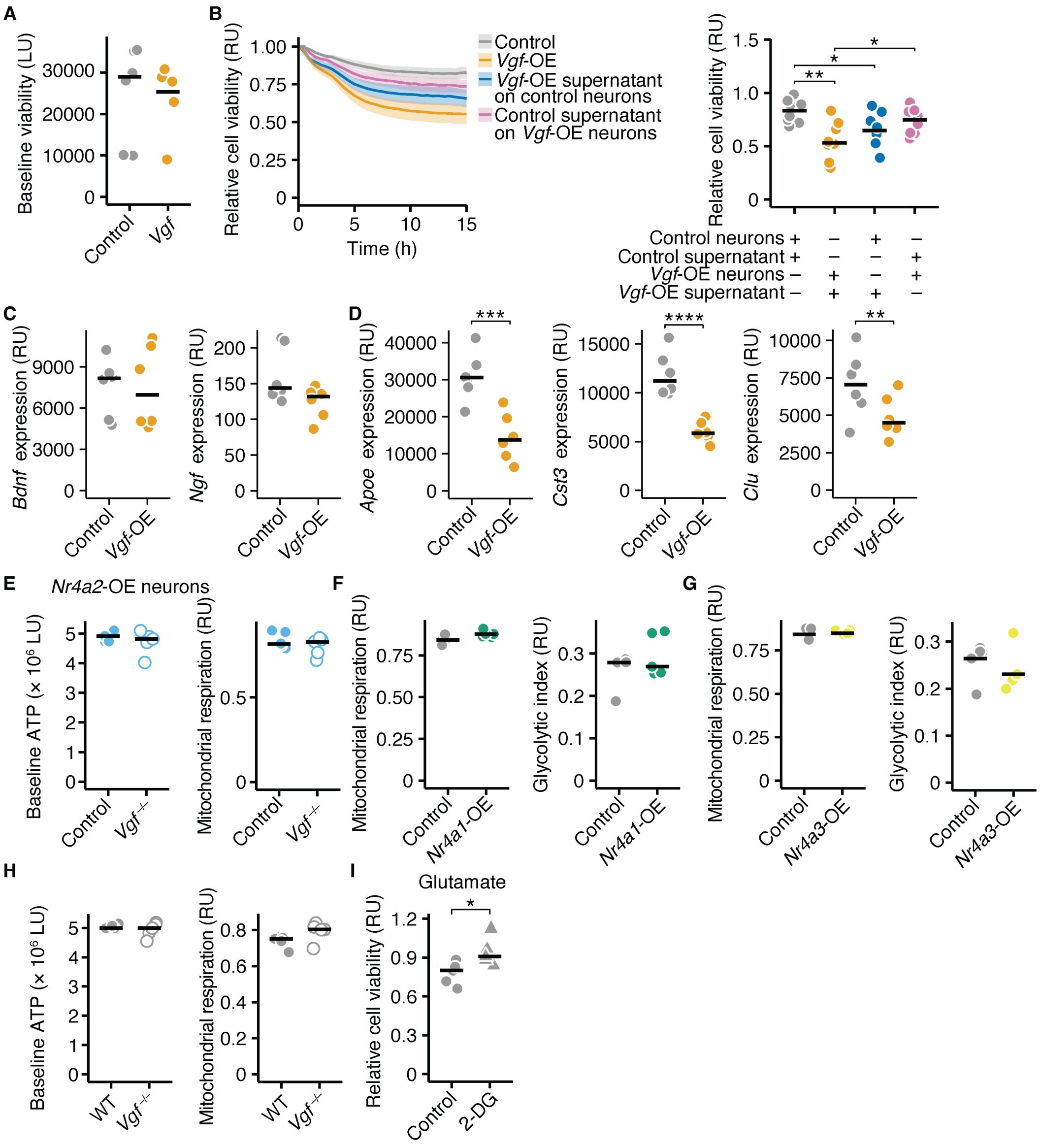

Supplemental Figure 4. Neurotrophic factors are not differently expressed in VGF-overexpressing neurons. (**A**) Baseline viability of control (GFP-) and VGF-overexpressing neurons (n = 6 per group). (**B**) Relative cell viability of EGFP (control) and *Vgf*-overexpressing neurons that were exposed to 50 μM glutamate for 15 hours. If indicated the medium of EGFP-overexpressing neurons was replaced with the medium of *Vgf*-overexpressing neurons and *vice versa* every other day (n = 8 per group). (**C and D**) Normalized expression counts in relative units (RU) of *Bdnf*, *Ngf* (C), *Apoe*, *Cst3*, and *Clu* (D) from our RNA-sequencing experiment of control (GFP-) and VGF-overexpressing neurons (n = 6 per group). Statistics were taken from differential expression analysis with FDR-correction for multiple comparisons. (**E**) Baseline ATP levels and mitochondrial respiration in NR4A2-overexpressing WT and *Vgf*-deficient neurons (n = 5 per group). (**F and G**) Mitochondrial respiration and glycolytic index in mScarlet- (control) and NR4A1- (F; n = 5 per group) or NR4A3-overexpressing neurons (G; n = 5 per group). (**H**) Baseline ATP levels and mitochondrial respiration in WT and *Vgf*-deficient neurons (n = 5 per group). (**I**) Relative cell viability (relative units = RU) of WT neurons that were exposed to glutamate and were pretreated with vehicle or 5 mM 2-DG (n = 5 per group). Points represent individual experiments, additionally mean is shown. If not stated otherwise, unpaired t-test with FDR-correction for multiple comparisons was used. $^*P < 0.05$, $^{**}P < 0.01$, $^{***}P < 0.001$, $^{****}P < 0.0001$.

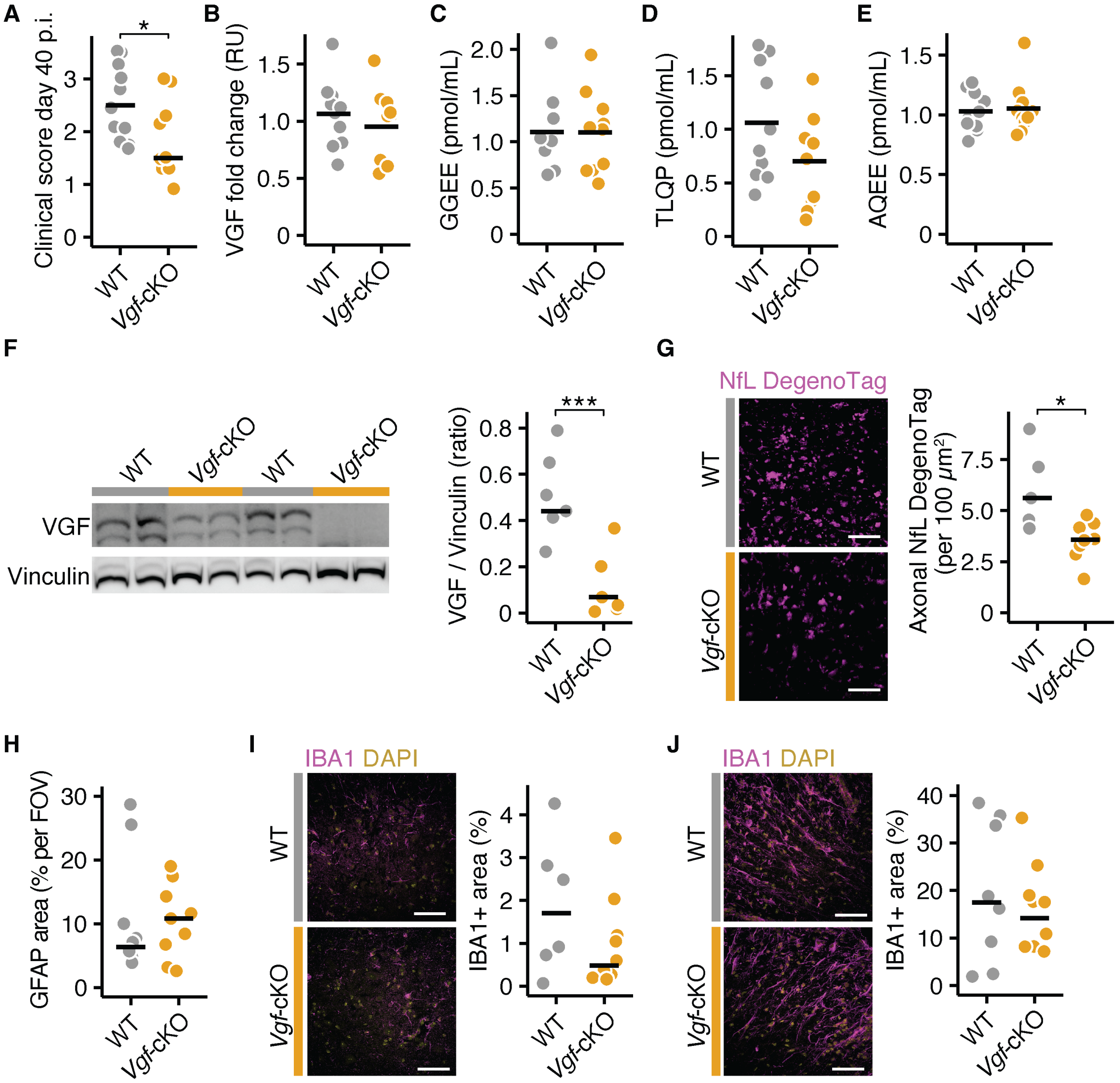

Supplemental Figure 5. Microglia activation is not different in *Vgf*-deficient and control EAE mice. (**A**) Clinical score at 40 days after immunization of WT (*n* = 11) and *Vgf*-cKO (*n* = 10) mice that underwent EAE. Kolmogorov-Smirnov test was used for statistical comparisons. (**B to E**) Relative fold change of blood levels of total VGF (B), GGEE (C), TLQP (D), and AQEE (E) in WT (*n* = 10), and *Vgf*-cKO (*n* = 10) EAE mice 40 day after immunization. Data were normalized to the average value of the WT animals. (**F**) Immunoblot analysis of brains of WT (*n* = 7), and *Vgf*-cKO (*n* = 7) EAE mice 40 days after immunization. The VGF protein levels were normalized to vinculin as loading control. (**G**) Number of NfL DegenoTag positive axonal puncta per 100 μm^2 in the dorsal columns of the cervical spinal cords of WT (*n* = 5) and Vgf-cKO (*n* = 8) EAE mice 40 days after immunization. Scale bar shows 10 µm. (**H**) Area covered by GFAP in *Vgf*-cKO (*n* = 9) and control mice (*n* = 10) undergoing EAE 40 days after immunization. Representative images of GFAP are shown in Figure 5F. (**I**) $IBA1^+$ covered area in the ventral horns of cervical spinal cords of WT (*n* = 6) and *Vgf*-cKO (*n* = 10) EAE mice 40 days after immunization. Scale bar shows 50 µm. (**J**) $IBA1^+$ covered area in the white matter of cervical spinal cords of WT (*n* = 8) and *Vgf*-cKO (*n* = 10) EAE mice 40 days after immunization. Scale bar shows 50 µm. Points represent individual experiments, additionally mean is shown. If not stated otherwise unpaired *t*-test was used for statistical comparisons. *$P < 0.05$.

SUPPLEMENTAL TABLES

Supplemental Table 3. Patients' demographics.

Control vs. relapse-remitting MS (RRMS)			
	n (% female)	Age, years, mean (SD)	Treatment, n (%)
Controls	20 (70)	30 (5)	NA
RRMS	20 (70)	30 (4)	0 (0)
Relapse vs. remission			
RRMS	7 (57)	34 (12)	0 (0)
Untreated RRMS vs. natalizumab-treated RRMS			
RRMS	6 (33)	36 (11)	6 (100)

Supplemental Table 4. Patients' characteristics for histopathology.

ID	Tissue	Acquisition	Phenotype	Age (years)	Sex
MS 1	Brain	Biopsy	MS	15	M
MS 2	Brain	Biopsy	MS	45	F
MS 3	Brain	Biopsy	MS	47	F
MS 4	Brain	Biopsy	MS	28	F
Control 1	Brain	Biopsy	Control	47	F
Control 2	Brain	Biopsy	Control	46	F
Control 3	Brain	Biopsy	Control	21	M
Control 4	Brain	Biopsy	Control	28	F

Supplemental Table 5. Primers and oligonucleotides used in the study.

Abbreviation	Name	Full sequence
Primer_f_1	for_mmVgf_KpnI	TAGGGTACCATG AAA ACC TTC ACG TTG CCG GC
Primer_r_1	rev_mmVgf_P2a_HindIII	TAGAAGCTT AGG ACC GGG GTT TTC TTC CAC GTC TCC TGC TTG CTT TAA CAG AGA GAA GTT CGT GGC TCC CGG GCG GTG CAG CAG CAC
Primer_f_2	for_mmNr4a1_BsiWI	TAG CGTACG ATG CCC TGT ATT CAA GCT CAA TAT GG
Primer_r_2	rev_mmNr4a1_XbaI	TAG TCTAGA GAA AGA CAA TGT GTC CAT AAA GAT CTT GT
Primer_f_3	for_mmNr4a2_AgeI	TAG ACCGGT ATG CCT TGT GTT CAG GCG CAG TAT
Primer_r_3	rev_mmNr4a2_XbaI	TAG TCTAGA GAA AGG TAA GGT GTC CAG GAA AAG TT
Primer_f_4	for_mmNr4a3_AgeI	TAG ACCGGT ATG CCC TGC GTG CAA GCC CA
Primer_r_4	rev_mmNr4a3_XbaI	TAG TCTAGA GAA AGG CAG GGT GTC AAG GAA GA

Supplemental Table 6. Antibodies used for VGF detection.

Antibody name	Antigen	Sequence	References	Antibody dilution	CV1%	CV2%
VGF Nt	h VGF $_{23–32}$	APPGRPEAQP	(5)	1:10,000	7	3
TLQP	h VGF $_{554–563}$	TLQPPASSRR	(6)	1:8,000	9	4
GGEE Nt.	h VGF $_{373–382}$	GGEERVGEED	(7)	1:20,000	5	2
GGGE Nt.	m VGF $_{375–384}$	GGGEDEVGEE	(8)	1:10,000	8	2
GAED Ct.	h VGF $_{408–417}$	EEDGEAGAED	(9)	1:150,000	8	3
NAPP	h VGF $_{485–493}$	NAPPEPVPP	(10)	1:40,000	7	4
AQEE	h VGF $_{586–595}$<br>m VGF $_{588–597}$	AQEEAEAEER	(6)	1:10,000	5	2

Abbreviations: Nt/Ct = N-terminus or C-terminus of the corresponding antigen sequence; h = human; m = mouse; CV1 = inter-assay coefficient of variability; CV2 = intra-assay coefficient of variability
