# Supplementary material for: The NR4A2/VGF pathway fuels inflammation-induced neurodegeneration via promoting neuronal glycolysis
Source: J Clin Invest. 2024 Jun 18;134(16):e177692. doi: 10.1172/JCI177692 (PMC11324305; doi:10.1172/JCI177692)

Full unedited gel for Supplemental figure 1F

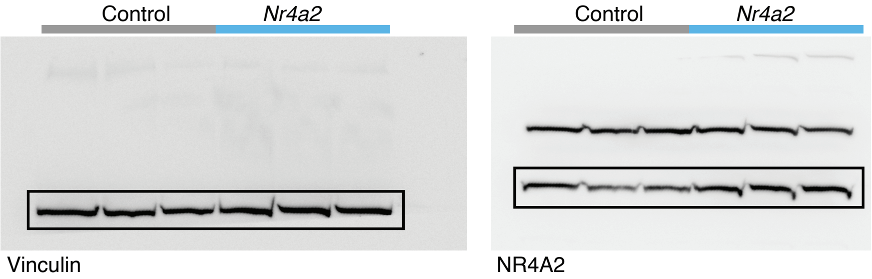

Full unedited gel for Supplemental figure 2F

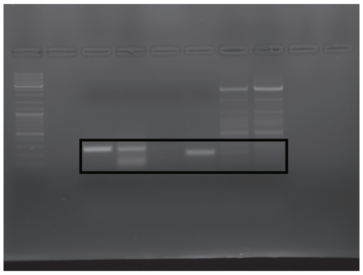

Full unedited gel for Figure 3B

EAE onset

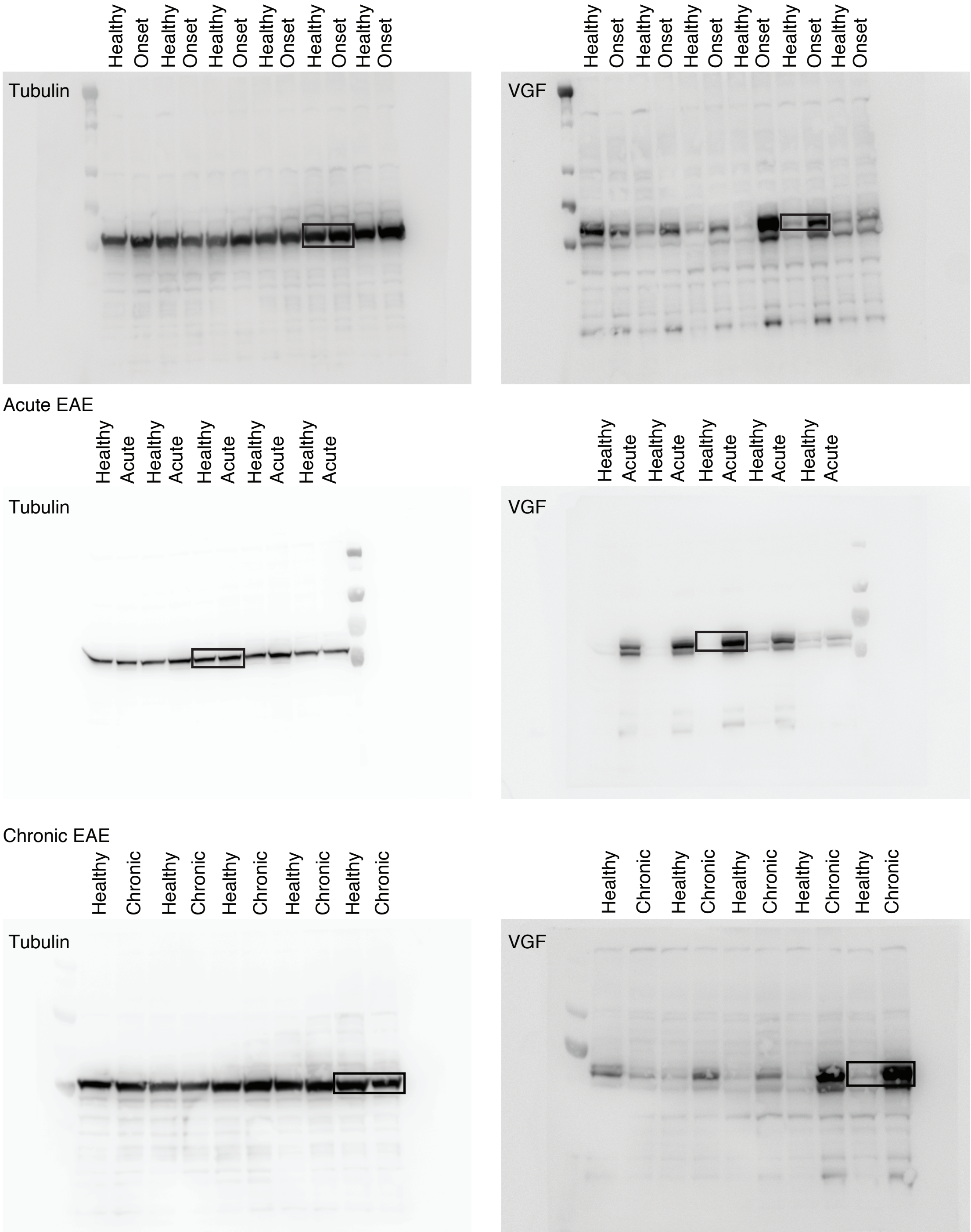

Full unedited gel for Supplemental figure 5E

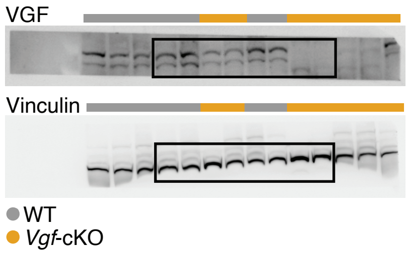

Supplement: Unedited blot and gel images [file jci-134-177692-s023.pdf]
